# AI‐Enabled Soft Sensing Array for Simultaneous Detection of Muscle Deformation and Mechanomyography for Metaverse Somatosensory Interaction

**DOI:** 10.1002/advs.202305025

**Published:** 2024-02-20

**Authors:** Jiao Suo, Yifan Liu, Jianfei Wang, Meng Chen, Keer Wang, Xiaomeng Yang, Kuanming Yao, Vellaisamy A. L. Roy, Xinge Yu, Walid A. Daoud, Na Liu, Jianping Wang, Zuobin Wang, Wen Jung Li

**Affiliations:** ^1^ Dept. of Mechanical Engineering City University of Hong Kong Hong Kong 999077 China; ^2^ Dept. of Electrical and Computer Engineering Michigan State University MI 48840 USA; ^3^ The Int. Research Centre for Nano Handling and Manufacturing of China Changchun University of Science and Technology Changchun 130022 China; ^4^ Dept. of Biomedical Engineering City University of Hong Kong Hong Kong 999077 China; ^5^ James Watt School of Engineering University of Glasgow Scotland G12 8QQ UK; ^6^ Sch. of Mechatronic Engineering and Automation Shanghai University Shanghai 200444 China; ^7^ Dept. of Computer Science City University of Hong Kong Hong Kong 999077 China

**Keywords:** human motion recognition, mechanomyography, natural human–machine interaction, non‐intrusive muscle activities sensing, wearable devices

## Abstract

Motion recognition (MR)‐based somatosensory interaction technology, which interprets user movements as input instructions, presents a natural approach for promoting human‐computer interaction, a critical element for advancing metaverse applications. Herein, this work introduces a non‐intrusive muscle‐sensing wearable device, that in conjunction with machine learning, enables motion‐control‐based somatosensory interaction with metaverse avatars. To facilitate MR, the proposed device simultaneously detects muscle mechanical activities, including dynamic muscle shape changes and vibrational mechanomyogram signals, utilizing a flexible 16‐channel pressure sensor array (weighing ≈0.38 g). Leveraging the rich information from multiple channels, a recognition accuracy of ≈96.06% is achieved by classifying ten lower‐limb motions executed by ten human subjects. In addition, this work demonstrates the practical application of muscle‐sensing‐based somatosensory interaction, using the proposed wearable device, for enabling the real‐time control of avatars in a virtual space. This study provides an alternative approach to traditional rigid inertial measurement units and electromyography‐based methods for achieving accurate human motion capture, which can further broaden the applications of motion‐interactive wearable devices for the coming metaverse age.

## Graphical abstract

A non‐invasive wearable device has been developed, incorporating a soft pressure sensor array that enables simultaneous detection of muscle deformation and mechanomyography. By leveraging machine learning techniques, this device demonstrates its ability to recognize a minimum of ten distinct lower limb motions, showcasing significant potential for future metaverse applications.

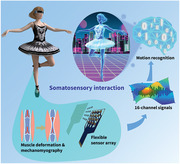

## 1 Introduction

With the rapid development of metaverse and human–machine interaction (HMI) applications, artificial intelligence (AI)‐enabled sensing technologies have attracted considerable attention.^[^ ^1^, ^2^, ^3^, ^4^, ^5^, ^6^ ^]^ Simultaneously, the need for developing devices and methods to detect human motion data (a crucial input in HMI systems) has surged in importance.^[^ ^7^ ^]^ In contrast to traditional vision‐based methods, the focus has shifted toward small and lightweight wearable devices that can overcome the limitations of high environmental requirements, such as the need for surrounding lighting, precise camera placement, and occlusion limits.^[^ ^8^ ^]^ Wearable devices based on inertial sensors, including accelerometers, gyroscopes, and inertial measurement units (IMUs), are commonly employed in both laboratory research^[^ ^9^, ^10^, ^11^, ^12^ ^]^ and commercial products (for example, the Apple Watch and the Fitbit smartwatch). The inertial sensors measure the acceleration and angular velocity of objects in motion across three mutually perpendicular axes. They can be placed on each joint to capture fine movements. However, their rigid nature makes them less user‐friendly, causing inconvenience to the users.^[^ ^13^, ^14^ ^]^ Recently, wearable human‐motion‐signal‐capturing devices based on soft pressure/strain sensors have captured considerable attention. Combined with machine learning, these devices exhibit the potential to facilitate human‐computer interaction.^[^ ^15^, ^16^, ^17^, ^18^, ^19^, ^20^, ^21^ ^]^ Notably, most research has focused on hand gestures when detecting gestures, often involving the use of stretchable flexible pressure/strain sensors on finger joints^[^ ^15^, ^18^, ^19^ ^]^ or the wrist.^[^ ^20^, ^21^ ^]^ As for lower‐limb motion detection, although gait recognition has been investigated by measuring the pressure distribution on the foot sole using triboelectric nanogenerators,^[^ ^16^, ^17^ ^]^ this approach is considered indirect. The detection and recognition of multiple human lower‐limb motions, especially those related to complex and large muscles, using soft pressure/strain sensors, have rarely been explored.

Muscle activities, such as stiffening, relaxation, and contraction, are integral to human motion. They are achieved through complex and highly coordinated mechanical interactions between bones, muscles, ligaments, and joints within the musculoskeletal system.^[^ ^22^ ^]^ Therefore, muscle activity measurement is a popular approach for capturing and recognizing human lower‐limb motions without interfering with joint movement. Presently, the standard muscle activity measurement techniques are electromyography (EMG)^[^ ^23^, ^24^ ^]^ and surface EMG (sEMG) with using noninvasive electrodes,^[^ ^13^, ^25^, ^26^, ^27^ ^]^ both of which measure the electrical signals generated by muscle contraction. These two techniques have demonstrated exceptional recognition accuracy for several common lower‐limb motions.^[^ ^24^, ^27^, ^28^, ^29^, ^30^, ^31^, ^32^, ^33^ ^]^ Moreover, EMG and sEMG have also been combined with inertial sensors to obtain more information.^[^ ^34^, ^35^, ^36^, ^37^ ^]^ sEMG, in particular, is more widely used than EMG due to its noninvasiveness and ease of use. Recently, in addition to traditional bipolar sEMG electrodes, multichannel sEMG devices with electrodes distributed in an array have been developed for applications in the detection of various human motions^[^ ^6^, ^38^, ^39^ ^]^ and gesture recognition systems.^[^ ^6^, ^39^ ^]^ Although EMG is the current standard muscle‐monitoring technology, it is characterized by a high hardware requirement, such as amplifiers to enhance weak electrical signals, and is sensitive to sweat secretion and changes in skin temperature. Its need for an electrical connection to the human skin and high cost make it less portable,^[^ ^40^, ^41^, ^42^ ^]^ limiting it applicability outside of laboratory settings. Mechanomyography (MMG), also called “muscle sound,” involves recording the low‐frequency lateral oscillation of muscle fibers during contraction, and is considered an alternative to EMG for muscle monitoring.^[^ ^43^ ^]^ MMG are characterized by low‐frequency vibrations (typically <50 Hz) that peak at ≈15–18 Hz, resulting in a skin surface displacement of ≈500 nm.^[^ ^44^ ^]^ MMG signals are primarily detected using piezoelectric contact sensors,^[^ ^45^, ^46^ ^]^ microphones,^[^ ^47^ ^]^ accelerometers,^[^ ^48^ ^]^ and laser distance sensors.^[^ ^49^ ^]^ Notably, MMG signals remain unaffected by sweat‐induced changes in skin impedance^[^ ^50^ ^]^ and do not require an amplifier,^[^ ^51^ ^]^ making them more versatile for various settings. They also have no specific sensor placement requirements.^[^ ^52^ ^]^ Furthermore, MMG signals provide valuable insights into changes in muscle force as they reflect the mechanical activities of muscles.^[^ ^43^, ^53^, ^54^, ^55^, ^56^, ^57^ ^]^ However, the broader applicability of MMG is limited by the absence of established dedicated sensors and the occurrence of vibrational/acoustic interference, often caused by environmental noise.^[^ ^57^ ^]^ Additionally, monitoring muscle shape changes induced by muscle contraction are valuable for motion monitoring.^[^ ^58^, ^59^ ^]^

**Figure 1:**
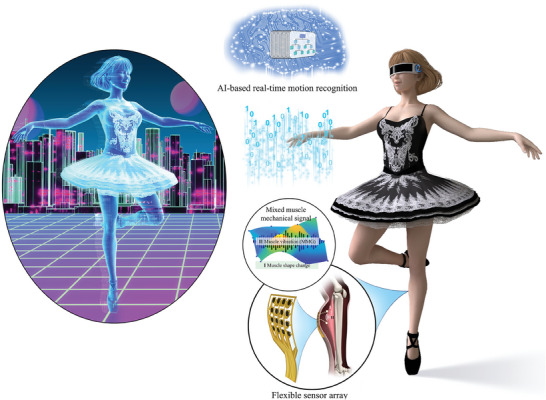
Muscle‐sensing device for lower‐limb motion recognition (MR)‐based human–computer interaction.

Muscle mechanical activities can be detected with a physical sensor. Therefore, we propose a soft pressure‐sensor‐based wearable muscle‐sensing device that simultaneously measures dynamic muscle deformation, that is, shape/morphology changes, and vibrational MMG signals associated with lower‐limb motion. Contraction of the calf muscles results in deforming the calf shape, keeping its volume almost unchanged.^[^ ^60^ ^]^ The resulting deformation of skin surface contours can be measured using wearable soft sensors. Moreover, the vibrational MMG signals induced by muscle fiber oscillations propagate to the skin surface and can be measured simultaneously. The device exhibits excellent repeatability for the same motion and exceptional specificity for distinguishing different motions. The obtained signals were analyzed and applied to recognize specific motions. Furthermore, a real‐time motion‐recognition system was developed and applied to realize somatosensory interactions in a virtual space. A conceptual illustration is presented in **Figure** **1**. This study also highlights the development of a novel soft sensor array with a wide bandwidth (0–60 Hz) for precise vibrational MMG measurements.

## 2 Results

### 2.1 Device Preparation

**Figure 2:**
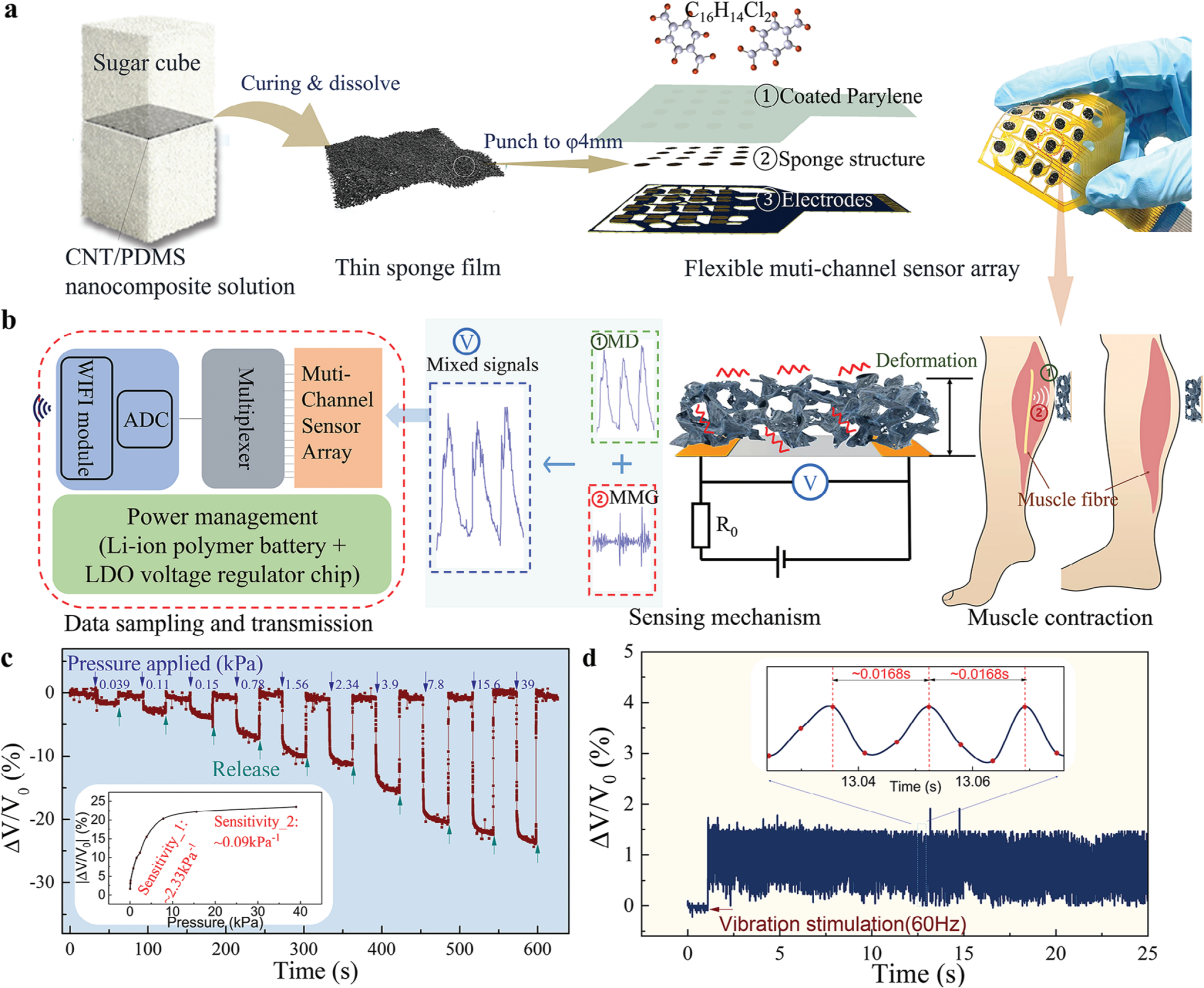
Design and working principle of the wearable muscle‐sensing device: a) Fabrication of the 16‐channel sponge‐based soft pressure sensing array, b) The signal generation and transmission mechanism of the soft sensing array‐based device (MD: Muscle deformation; MMG: Mechanomyography), c) Response of an individual sensory element to different applied static pressure (Inset: The pressure sensitivity derived), and d) Response of the sensory element to vibration simulation at 60 Hz (Inset: The enlarged view).

The entire device comprised a 16‐channel sponge‐based pressure sensor array with a sensing area of ≈32 mm × 28 mm, connected to an ESP32‐based wireless signal (WIFI protocol) acquisition/transmission circuit board (**Figure** **2**). The sensor array consists of 4 × 4 elements with a carbon nanotube (CNT)/polydimethylsiloxane (PDMS) sponge structure (Figure 2a). The detailed fabrication processes of the sponge structure and electrode substrate design are presented in Figure S1 and Text S1 (Supporting Information). This sensing element exhibited high sensitivity with a wide bandwidth capability (that is, input stimulus ranging from static pressure input to vibration input of up to hundreds of Hertz) for skin movement/vibration detection, as reported in our previous study.^[^ ^61^ ^]^ The sensor array is highly flexible and conformable to the human skin due its hollow‐carved design. Each element functions independently, wherein each sensing element is decoupled from the other elements. The sensing mechanism and signal transmission from the sponge‐structured sensory elements are illustrated in Figure 2b. Muscle movement (including muscle shape change and vibrational MMG) induces the deformation of sponge‐based sensing elements, which function as piezoresistive pressure sensors, inducing a change in the divided voltage across the sensor. The change in the dynamic contact pressure between the skin surface and sensing elements was detected by the sensor array. The sensor response is measured using a simple voltage divider. The sampling rate of each sensing element was ≈120 Hz, with a sampling interval of ∼0.5 ms for each sensing channel. This sampling rate can capture all human motions, in accordance with the Nyquist sampling theorem, as the frequency of human activities typically ranges from 0–20 Hz, with 98% of human activities featuring a frequency of <10 Hz.^[^ ^62^ ^]^ Moreover, this sampling rate effectively covers the vibrational MMG signals, whose power spectrum mainly ranges from 10 to 50 Hz, with a dominant frequency below 30 Hz^[^ ^63^, ^64^ ^]^ and a peak at ≈15–18 Hz.^[^ ^44^ ^]^ The response of individual sensory elements are depicted in Figure 2c, demonstrating that the rate of change in the output voltage increased with applied pressure. These sensing elements reached a sensitivity of ∼2.3 kPa^−1^ in the pressure range of 0.03–7.8 kPa, while the sensitivity decreased to ∼0.09 kPa^−1^ in the pressure range of 7.8–39 kPa. The voltage value (*V*) represents the output signal. The sensitivity performance (within the pressure range of 0.03–7.8 kPa) for all 16 channels in a sensor array was illustrated in Figure S2 (Supporting Information), with an average value of 2.34 ± 0.27 kPa^−1^. Figure 2d showcases the ability of the sponge‐structured sensor to detect vibrations at 60 Hz. Therefore, the dynamic muscle activity induced by human lower‐limb motion can be recorded using the sponge sensor array.

### 2.2 Human Motion Detection with the Muscle‐Sensing Device

**Figure 3:**
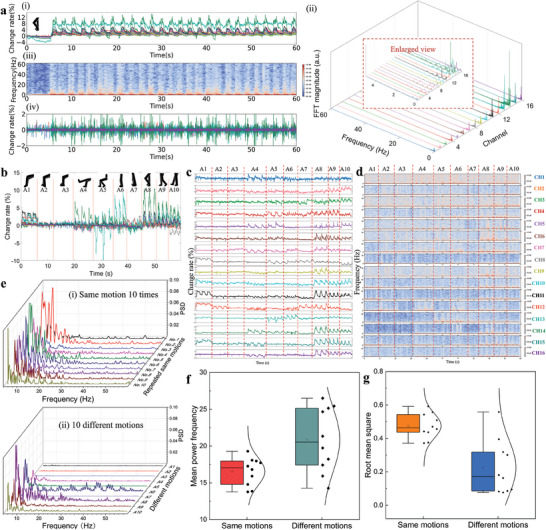
Response of the soft sensing array to different human lower‐limb motions: a(i)) Original signals of a piece of continuous stepping motion (A8), ii) Fast Fourier transform (FFT) of the signals. (a.u., arbitrary units) Inset: Enlarged view of the 0–5 Hz range, iii) Short‐time Fourier transform (STFT) of the signals, and iv) Signals subjected to high pass filtering at 5 Hz. b) Time response of the sensing array to multiple motions (A1–A10); c,d) Amplitude, and frequency response of each channel to the ten motions. Investigation of high‐frequency (that is, >5 Hz) MMG signals; e) Power spectral densities (PSDs) of i) the same motions for ten cycles and ii) ten different motions of MMG signals; f) Boxplot of statistics on the mean power frequency (MPF) of MMG signals; and g) Boxplot of statistics on the mean root mean square (RMS) of the MMG signals of the same motions and different motions (A1: heel lift, A2: toes lift, A3: foot inversion, A4: forward leg stretch, A5: backward leg stretch, A6: standing with foot inversion, A7: turning around, A8: stepping, A9: forward walk, A10: backward walk).

The constructed wearable muscle‐sensing device is flexible and portable due to the hollow‐structure electrodes, soft sponge sensing materials, and convenience in gathering muscle‐activity signals via wireless data transmission. The device was employed to detect 10 types of lower‐limb motions: sitting motions of (A1) heel lift, (A2) toes lift, (A3) foot inversion, (A4) forward leg stretch, (A5) backward leg stretch, and standing motions of (A6) standing with foot inversion, (A7) turning around, (A8) stepping, (A9) forward walk, and (A10) backward walk. The corresponding images of these motions are shown in Figure S3 (Supporting Information). These motions include muscle training activities (such as heel lift, toe lift, and forward leg stretch), normal/daily walking activities (including stepping and forward walking), and abnormal motions (for example, foot inversion) that may cause injury. The flexible sensor array was directly attached to the skin of the subjects around the calf muscle (Figure S3, Supporting Information), with the circuit box connected to the sensing array affixed to the leg using an elastic strap to ensure firmness during specified actions. **Figure** **3** depicts the output signals of different lower‐limb motions when testing the device on a typical human subject. Figure 3a(i) displays the change‐rate response of the 16 channels for a repeated stepping motion (A8) for 60 s, and the corresponding fast Fourier transform (FFT) is presented in Figure 3a(ii). The FFT spectra of the signals obtained over the entire motion time featured peaks below 1 Hz, which mainly reflected the human‐controlled frequency for lower‐limb motions. A short‐time Fourier transform (STFT) spectrogram of Channel 14 is depicted in Figure 3a(iii), which highlights some high‐frequency components of the obtained signals. The response of channel 14 was chosen as the representative for the STFT because it exhibited the most significant change overall; the STFT plots of all 16 channels are displayed in Figure S4 (Supporting Information). The high‐frequency components (particularly >10 Hz) mostly corresponded to the motion start period, indicating that the high‐frequency components were mainly induced by muscle motions. During muscle contractions, mechanical vibrational signals (that is, MMG) are generated at a low cutoff frequency of 1–2 Hz.^[^ ^53^ ^]^ Therefore, the obtained motion signals were considered to comprise MMG signals of muscle activity. To further investigate the MMG signals, a 5‐Hz high‐pass filter was applied to the time‐domain signals to remove the lower‐frequency signals because this frequency threshold (5 Hz) was commonly used in previous research to isolate pure MMG signals.^[^ ^65^, ^66^ ^]^ Figure 3a(iv) shows the filtered time‐domain signals (MMG signals) of the calf muscles for motion A8, where the signal amplitude (that is, the value of the change rate) substantially decreased compared to the original signals. This reduction implies that the large‐amplitude signals were primarily caused by muscle shape changes, which have a human‐subject‐controlled motion frequency (<1 Hz).

The responses of all 16 channels to the ten motions are provided in Figure 3b, and the corresponding FFT plot is presented in Figure S5 (Supporting Information). Each channel responds differently to these motions. Small movements, such as foot inversion, induce a smaller response because they involve weaker muscle motion. Large movements, such as stepping, generate larger responses due to the involvement of larger/more significant muscle motions. The high resolution achieved by the multichannel sensor array is beneficial for distinguishing between similar leg motions. For example, the stepping (A8), forward walking (A9), and backward walking (A10) muscle motions are quite similar. Although the three motions caused the greatest change in channel l4 and exhibited similar waveforms, the different output signals in the remaining channels and corresponding combinations of the 16 channels allowed us to distinguish the three motions. The amplitude and frequency responses of the signals (obtained via the STFT) collected from each channel are shown in Figure 3c,d, respectively. The standard deviations of the output signal change rates of the stationary and in‐motion states of A1–A10 were calculated and compared (Figure S6, Supporting Information). The output signals experienced only slight changes in the stationary state (indicated by black symbols) due to various environmental noises. However, they changed substantially during motion (represented by red symbols), indicating that each channel functioned well and played a role during the test. MMG signals are typically linked to muscle force, which can be used in many fields. Therefore, further investigation was performed to prove the effectiveness of the MMG signals acquired in this study for future applications. The power spectral density (PSD) was calculated to display the spectral energy distribution over the frequency, which reflected the signal frequency feature. The PSD curves of the filtered MMG signals of the same motion for ten cycles (Figure 3e(i)) were almost equal, whereas the PSD curves of the 10 different motions (Figure 3e(ii)) differed significantly. Two crucial parameters of MMG related to the frequency and magnitude of muscle contractions were also examined, namely mean power frequency (MPF) (Equation (1)) and root mean square (RMS) (Equation (2), respectively.^[^ ^57^ ^]^ Figure 3f,g presents boxplots of the MPF and RMS for signals generated during ten cycles of the same motion and ten different motions. The MPF and RMS for the same motion were more concentrated, while those produced by different motions were more dispersed. In summary, these results demonstrate that the developed muscle‐sensing device can detect both dynamic muscle shape changes and vibrational MMG signals during human lower‐limb motions. The device exhibited excellent repeatability for the same motion and high level of specificity for different motions, rendering it promising for human‐motion tracking, recognition, and related applications.

### 2.3 Recognition of Lower‐Limb Motions and Gait Identification

**Figure 4:**
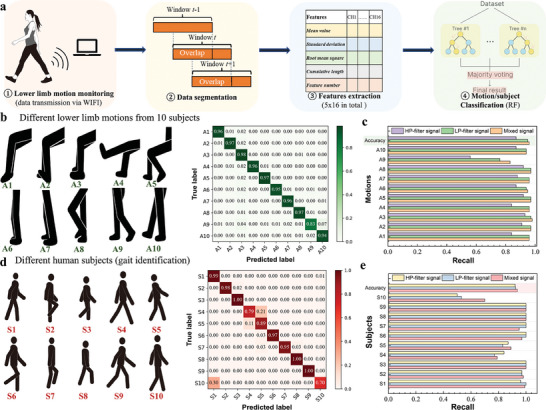
Recognition of 10 lower‐limb motions and gait identification of 10 human subjects: a) Data process flow. RF: random forest. Classification results of lower‐limb MR (motions A1–A10) and gait identification (subjects S1–S10). b,d) Confusion matrix of MR, and gait recognition. c,e) Recognition accuracy comparison of using the high‐pass filtered signal (>5 Hz), low‐pass filtered signal (<5 Hz), and the mixed signal for MR, and gait recognition.

The recognition of specific lower‐limb motion recognition (MR) holds considerable application potential in health monitoring, rehabilitation, assistive robotic control, and game entertainment. As demonstrated above, the proposed sensing device can provide rich motion information regarding the muscle mechanical activity. The acquired signals were processed to realize MR, as shown in **Figure** **4**a. Ten human subjects were recruited to test the device developed for lower‐limb MR. Basic information regarding the age and body mass index (BMI) of all participants is provided in Figure S7 (Supporting Information), which suggests that they fall within the age range of 22–30 y and all have underweight or healthy weight according to their BMI values. The collected data were segmented into 4‐s fixed windows, and sliding windows with different overlaps were investigated. For each window, five time‐domain features (Equations S4∼S7, Supporting Information), including the mean value, standard deviation, root mean square, cumulative length, and peak number of each channel were calculated and used as the input feature matrix for the random forest (RF) algorithm. The RF method combines multiple randomized decision trees and aggregates their predictions for achieving the final classification results, showing excellent performance in tasks with numerous variables.^[^ ^67^ ^]^ Additionally, recursive feature elimination with five‐fold cross‐validation (RFECV) was used for optimal feature number selection. Figure 4b illustrates the classification results of the ten motions collected from the ten subjects using a confusion matrix (fixed window). The cross‐validation accuracy, with an average of ≈96.06%, is shown in Figure S8a (Supporting Information). The optimal number of features was determined to be 59, and the model consistently generated comparable accuracies for different validation sets. The sensor array was designed to comprise 16 channels (4 × 4) in this study, and the effect of the number of array channels on the MR‐performance was further investigated. As depicted in Figure S9 (Supporting Information), the recognition accuracies of the 2 × 2 and 3 × 3 arrays (extracted from the original 4 × 4 array) were compared. The results of 87.94%, 94.15%, and 96.06% for using 2 × 2, 3 × 3, and 4 × 4 array, respectively, suggest that the MR‐accuracy increases as the channel number increases. The classification results for the different sliding window overlaps are listed in Table S1 (Supporting Information). These results highlight that high‐precision MR can be achieved using the developed wearable muscle‐sensing device with the RF as the classification algorithm. MR‐accuracy reached ≈99.4% when an 80%overlapping sliding window was applied. Figure S10 (Supporting Information) demonstrates the MR results (with 4‐s fixed window segmentation) based on the individual datasets of the ten subjects respectively, with the recognition accuracy of each subject exceeding 94%. Figure 4c displays the recognition accuracy achieved when using the high‐pass filtered signal (> 5 Hz), low‐pass filtered signal (<5 Hz), and the original mixed signal for MR. The results indicate that both low‐frequency signals (macrodynamic muscle‐shape change) and high‐frequency signals (vibrational MMG) contribute to MR; thus, using the combined original signals achieves the best accuracy.

Gait is considered a personalized biological feature, similar to fingerprints, iris patterns, face shapes, and voice acoustics.^[^ ^17^ ^]^ One notable advantage of gait as a biometric identifier is that it is observable from a distance.^[^ ^68^ ^]^ The calf muscles, which contribute to the ankle plantar flexor moment and generate a knee flexion moment during walking, play a critical role in indicating healthy and abnormal gait stance phases.^[^ ^69^ ^]^ Therefore, the developed wearable muscle‐sensing device (attached on the calf muscle) can be used for gait analysis and identification tasks by measuring calf muscle mechanical activity. Ten participants wore muscle‐sensing devices and walked at self‐selected natural speeds. The results obtained from the gait data are presented in Figure 4 d,e and Figure S8b (Supporting Information). The recognition accuracy for ten subjects reached 93.79%, with an optimal feature number of 60. A comparison of the effects of different sliding window overlaps on gait identification (Table S2, Supporting Information) revealed that a larger overlap corresponded to a higher recognition accuracy, reaching 96.83% with an 80% overlap sliding window. The results underscored the potential applicability of the developed device for biometric (gait) identification. Compared with MR, the classification accuracy relationship of using the HP filter, LP filter, and mixed signals is slightly different for gait identification. Although the original mixed signals obtain the highest accuracy, the accuracy of using LP‐filter signals is more similar to that of mixed signals and significantly higher than that of HP‐filter signals for MR, while the accuracy of the LP‐filter signals is closer to that of the HP‐filter signals and evidently lower than that of the mixed signals for gait identification (Figure S11, Supporting Information). This might be because the time‐dependent muscle deformation pattern (e.g., Figure 3b) significantly differs among different motions, while the basic gait cycle, consisting of two phases (stance and swing phase^[^ ^70^ ^]^), is similar for each subject. Therefore, the LP‐filtered muscle deformation information is much more critical for MR, resulting in an accuracy identical to that of the original mixed signals. Although all human gaits generally comprise two phases, the specific parameters for each individual are unique.^[^ ^71^ ^]^ For example, the calf muscle force (that is highly related to MMG signals), which helps people maintain stability and forward propulsion, is a crucial gait parameter.^[^ ^72^ ^]^ Therefore, both LP‐filtered and HP‐filtered information are important, with the mixed signals achieving the highest recognition accuracy. To further investigate the effect of walking speed on gait identification, two subjects (S1 and S3) were asked to perform additional experiments involving walking at speeds faster and slower than their natural speed while wearing the developed device (Figure S12a, Supporting Information). Subsequently, the gait data at different speeds were recognized with the model trained in Figure 4d (that is, the model trained with data of only natural walking speed). The results showed that the recognition accuracy can decrease if the walking speed varies (Figure S12b, Supporting Information), However, the accuracy could be improved by adding varied walking data to the model training dataset (Figure S12c, Supporting Information). This suggests that different walking speeds should be considered when constructing a more general and robust gait‐identification model for practical applications.

### 2.4 Real‐Time MR System for Somatosensory Interaction Application

**Figure 5:**
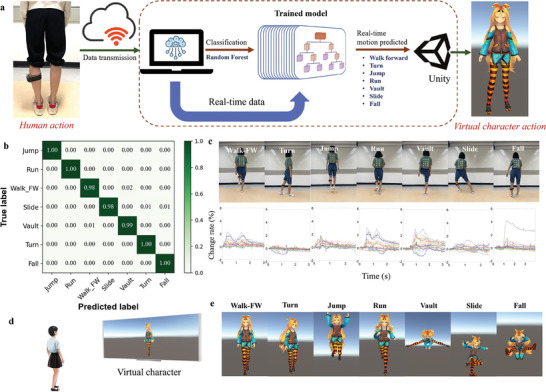
A real‐time MR system and MR‐based human–computer interaction application with the wearable device. a) Process flow of the somatosensory interaction with virtual space. b) Confusion matrix produced by the trained MR model for real‐time classification and prediction. FW: forward. c) Different human motions. and the corresponding output signals produced by the proposed device. d) Experimental set up. e) The avatar motions controlled by the human motions in virtual space.

Gesture and motion control are common interaction methods for video games with numerous advantages (e.g., physical and emotional engagement, overweight and obesity alleviation, and encouragement of social interaction) compared with the traditional keyboard‐and‐mouse approach,^[^ ^73^ ^]^ which also plays an important role in the coming metaverse age. As the offline motion‐recognition capacity of the device was proven, the real‐time motion‐recognition capacity of the device was investigated. Here, we present a simple demonstration of controlling the motion of a virtual character by providing information about the tester's lower‐limb muscles. A virtual scene was constructed using the Unity cross‐platform game engine. Human‐activity signals were sampled using a wearable muscle‐sensing device. Real‐time data were transmitted to the computer and fed into the trained MR model, and the predicted motion command was sent to Unity to control the virtual character (**Figure** **5**a). First, a dataset consisting of signals of seven lower‐limb motions (that is, forward walking, turning, jumping, running, vaulting, sliding, and falling) was created. Subsequently, a trained MR model was built based on the dataset using an RF algorithm with RFECV, as performed for the offline recognition of the abovementioned ten motions. However, to reduce the online computational load, the original 16 channels were fused into eight channels before feature extraction. The fused feature matrix consisted of 40 features (that is, 5 × 8) for each window. The training result is shown in Figure 5b with the accuracy of ∼99.45%. The output signals for the seven motions are shown in Figure 5c. Finally, when the human subject performed any of the seven motions, the output signals were transmitted to a computer and fed into the trained model (Figure 5d). The virtual character performs the corresponding motion according to the predicted results (Figure 5e). The real‐time motion control system is shown in the Supplementary Movie. This demonstrates the potential applications of the developed wearable muscle‐sensing device in somatosensory games and virtual reality interfaces.

## 3 Discussions

Over the past decades, various techniques and sensing devices have been developed for human motion capture and recognition, particularly with the emergence of smart wearable devices such as smartwatches and smart wristbands. The advantages and disadvantages of several commonly used human motion sensing devices with different measurement principles are listed in Table S3 (Supporting Information). Compared to other devices, muscle sensors can provide information on muscle function and illustrate dynamic motions from the perspective of muscle activities, which is an additional advantage to the most commonly used IMU‐based wearable devices. Muscle contraction is accompanied by muscle shape changes, which allow the measurement of induced psychological electrical and mechanical vibration signals on the skin surface.^[^ ^66^ ^]^ Several common methods have been developed for monitoring muscle activity based on electrical and mechanical signals such as EMG and MMG. Compared to EMG/sEMG, MMG is characterized by good robustness, portability, and does not require an amplifier. However, there is a lack of dedicated sensors for MMG measurements, and condenser microphones and accelerometers are mostly used for measurements (Table S4, Supporting Information). Muscles can also be monitored according to morphological changes in muscle shape during muscle contraction (that is, muscle fibers shorten in length and increase in circumference to maintain volume).^[^ ^60^ ^]^ One study reported a fiber strain sensor for estimating the muscle force through muscle circumference measurements.^[^ ^59^ ^]^ Researchers have also applied capacitive sensors to measure muscle shape changes using the human body as a dielectric layer.^[^ ^74^ ^]^ However, these muscle‐shape measurement methods are not mature or convenient enough for wide application. With the development of advanced materials and fabrication techniques in recent years, flexible pressure/strain sensors with various working mechanisms have been proposed to be attached to the human skin surface conformably for human body motion detection; several typical works are listed in Table S5. Compared with other works, the muscle‐monitoring device based on the flexible sensor array developed in this work features the simultaneous detection of muscle morphology/shape deformation and muscle vibrational MMG signals. This rich and reliable information helps to achieve high recognition accuracy (that is, 96.06%) by testing on multiple (that is, 10) human subjects for ten lower‐limb motions and enables human–machine interaction. The muscle sensing device developed in this work can detect muscle sounds (that is, MMG signals) because the sponge‐structured pressure‐sensing elements have been demonstrated to have a wide frequency detection range that can cover the acoustic sound range in our previous work.^[^ ^61^ ^]^ Compared to other flexible wide‐bandwidth sensor,^[^ ^75^, ^76^, ^77^, ^78^, ^79^, ^80^ ^]^ the sponge structured sensor can be fabricated using a convenient and cost‐effective method (that is, sacrificial sugar template), and the piezoresistive working mechanism enables a simple circuit design. In addition, the design of the multichannel array would increase the information quantity and reliability, which is beneficial for MR applications (Figure S9, Supporting Information). This has also been reported in previous research on multichannel sEMG with array‐distributed electrodes for MR applications.^[^ ^39^ ^]^ Because recent studies on flexible pressure/strain sensors are rarely used for human lower‐limb MR, a comparison of the lower‐limb motion‐recognition performance of some reported EMG/sEMG‐based devices and the proposed device is shown in Figure S13 (Supporting Information). The studies shown in Figure S13 (Supporting Information) mainly used bipolar sEMG electrodes; therefore, usually more than one sensors are applied for MR. Array‐distributed multichannel sEMG electrodes have been developed for human motion detection with one piece of array covering a portion of the skin surface in recent years, but only gesture recognition systems based on them have been reported for now as far known as the authors^[^ ^6^, ^39^ ^]^ so this type of sEMG was not considered in Figure S13 (Supporting Information).

As both muscle shape changes and MMG signals are related to muscle strength, the signals obtained from the proposed wearable device can be used for muscle strength estimation. The output signals of the leg lift motion under different leg weights (Figure S14, Supporting Information) and forearm muscle static contractions (Figure S15, Supporting Information) can be used to roughly map the output signals with muscle strength. However, muscle force estimation is a complex process, and the theoretical discussion is presented in Text S2 (Supporting Information). Further experiments can be conducted to investigate the more detailed relationships between the output signals and muscle strength. Nevertheless, as different muscle forces can cause different output signals, the effect of muscle strength during MR was investigated by asking the human subject to perform the same motions while wearing a weight on the ankle. As shown in Figure S16a (Supporting Information), the accuracy of recognizing ten different weight‐free motions of S1 (that is, no weight‐bearing) was ≈99.29%. Subject S1 was then asked to perform the same ten motions while wearing a 2 kg sandbag weight on the ankle. The weight‐bearing motion signals were then recognized with the same model (that is, the model trained with weight‐free motion data), and the accuracy was ≈87.29% (Figure S16b, Supporting Information). Similarly, gait identification accuracy (natural walking speed) may also decrease when the subjects walk while wearing a weight on the ankle (Figure S16c,d, Supporting Information). A potential solution to this issue is to improve the universality of the recognition model by increasing the training dataset with motion data under different conditions, as shown in Figure S16e,f (Supporting Information), which uses improved models by adding weight‐bearing motion/gait signals to the model training dataset. In addition, although the ten participants in the experiments all have a underweight or healthy weight according to BMI values, the effect of body fat, including intramuscular^[^ ^81^ ^]^ and subcutaneous^[^ ^82^ ^]^ fat, on mechanical muscle activity detection is discussed in Text S3 (Supporting Information). Body fat affects muscle bulging and attenuates mechanical waves; therefore, the sensing performance of the sensor would be degraded in overweight people, similar to sEMG.^[^ ^83^ ^]^ Therefore, further improvements may be necessary when applying the developed device to overweight or obese individuals.

In MMG applications, motion artifacts are image disturbances with a human‐subject‐controlled frequency induced by the subject's body movements.^[^ ^66^ ^]^ Motion artifact signals are usually introduced when accelerometers are used; therefore, filtering is necessary to obtain the “real” MMG signals. The frequencies of the limb/body motions may overlap with the MMG signal frequencies. Condenser microphones are optimal sensors for measuring MMG signals in muscle contraction experiments because they are not sensitive to motion artifacts. However, motion artifact signals also contain useful information about human motion and play an important role in kinematic‐related applications. For example, MMG sensors have been used with an IMU to investigate muscle activity during limb motion.^[^ ^57^, ^84^, ^85^ ^]^ Although the muscle length and the tissue thickness between the muscle and the MMG sensor can affect MMG signals, there is convincing evidence that the MMG signals can provide valid information on muscle functions for dynamic muscle actions.^[^ ^86^ ^]^ The flexible sensor array developed in this study detected both subject‐controlled motion signals and muscle fiber vibration‐controlled MMG signals. Although relatively clean MMG signals can be obtained using a low‐pass filter owing to the very low subject‐controlled motion frequency (that is, mostly <1 Hz), filtering is not necessary for the MR application discussed in this study, and so‐called motion artifacts are desired in this investigation. Therefore, a low‐pass filter was not applied during data processing for motion classification and human–machine interaction.

## 4 Conclusion

In conclusion, a wearable AI‐enabled muscle‐sensing device was developed for human lower‐limb motion monitoring. Real‐time multi‐MR and the application of HMI were demonstrated. A soft sensor array can measure dynamic muscle shape/morphology changes and vibrational MMG signals during muscle contractions. The multichannel design offers high spatial resolution and rich information. The 16‐channel sensor array was designed with suspended and decoupled mechanical sensing elements, each of which was fabricated with an ultrathin CNT/ PDMS nanocomposite‐based sponge and exhibited a wide detection bandwidth. With fixed window segmentation, ten lower‐limb motions were recognized, with an offline classification accuracy of ≈96.06% using an RF algorithm. The gait data were further applied as biometric parameters for 10‐subject motion identification with an accuracy of ∼93.79%. Finally, the developed device enabled somatosensory virtual reality interactions for real‐time control of a metaverse avatar performing seven motions. The proposed wearable muscle‐sensing device and corresponding real‐time MR system have prospective applications in smart wearables.

## 5 Experimental Section

### Device Preparation

The sensing elements of the sensor array were fabricated using a CNT/PDMS nanocomposite material synthesized using isopropyl alcohol (IPA) as the solvent, as described in Text S1 and Figure S1a,b (Supporting Information). After the ultrathin sponge structure was obtained, individual round sensory elements with a size of φ4 mm × 400 µm were then punched out. The flexible substrate with a thickness of ∼150 µm was fabricated with polyimide as the structural material, which covered a thin gold layer as the electrical connector at the electrode positions. The detailed design of the electrode substrate is shown in Figure S1c (Supporting Information), which was then processed by a professional company (Shenzhen Phaeton Xin Electronics Co., LTD, China) using flexible polyimide copper‐clad laminates. Individual sponge sensory elements were attached to each electrical connector of the flexible substrate using silver ink. Finally, a 30‐nm thick Parylene‐C layer was coated on the surface of the entire sensor array. The coating process was applied under 17 millitorr, the pyrolysis temperature was 690 °C, the vaporization temperature was 175 °C, and the deposition temperature was room temperature. The resulting thickness was ≈30 nm when 0.1 g Parylene‐C was applied. A simple voltage divider circuit was designed using a feature‐rich MCU ESP32 board. The output voltage signal of the 16‐channel sensor array was acquired using a 16‐channel multiplexer. The acquired data were transmitted to a computer via a WIFI protocol at a sampling rate of ≈120 Hz for the whole 16 channels. The entire device was powered by a chargeable Li‐ion polymer battery, and a low‐dropout regulator chip, MCP1725T, was used to provide a stable 3.3‐V voltage.

### Experiments on Human Subjects

Ten human subjects wearing the prepared wearable muscle‐sensing device were recruited to perform ten lower‐limb motions. The ten motions included five sitting motions (foot inversion, heel lift, toe lift, forward leg stretch, and backward leg stretch) and five standing motions (standing with foot inversion, foot inversion, turning around, stepping, forward walking, and backward walking). The sponge‐based sensor array was attached to the calf (mainly covering the gastrocnemius) and the upper edge of the sensor array is ≈3 cm away from the knee using a 3M Tegaderm HP Transparent Film Dressings with the size 10 cm × 12 cm. The sensor was placed in the middle of the 3 m film, which was kept flat without stretching when it adhered to the skin surface. The average circumference of the calf (measured at 3 cm below the knee) of the participating human subjects is 33.78 ± 2.41 cm. The data acquisition and transmission boards were fixed to the legs using elastic straps. The subjects performed each motion repeatedly at a frequency of ≈0.5 Hz and for 90 s each time. Each subject performed four sets of each motion, with a minute of rest between sets. For forward and backward walking, the subjects walked along a ∼10 m straight indoor passage at a self‐selected speed 15 times back and forth. The experiments were conducted indoors with stable Wi‐Fi coverage and the data were obtained using a laptop. The experiments were approved by the Human Subjects Ethics Sub‐Committee of the City University of Hong Kong (Reference NO. 2‐2022‐62‐F), and written informed consent was obtained from all participants.

### Data Processing and Offline Classification

The change rate (i.e., *X*  =  (*V* − *V*_0_)/*V*_0_ × 100%) of the output voltage signal was first calculated for baseline adjustment. The MPF was calculated from the PSD according to Equation (1):

(1) $$MPF=\frac{\sum_{i\;=\;1}^{N}f_{i}*P_{i}}{\sum_{i\;=\;1}^{N}P_{i}}$$

where *f_i_* is the frequency, *P_i_* is the PSD value at frequency *f_i_*, and *N* is the data number. The variance was calculated using Equation (2):

(2) $$\mathrm{Root}\;\mathrm{mean}\;\mathrm{square}:RMS=\sqrt{\frac{1}{N}\sum_{i\;=\;1}^{N}X_{i}^{2}}$$

For offline motion recognition and gait identification, the data were then segmented into 4‐s sliding windows. The total sample sizes for the motion and gait identification are 8304 and 379, respectively. The time‐domain average, root mean square, standard deviation, and cumulative length of the signal of each channel for each window were calculated as a part of the feature matrix using Equation (2) and Equations S5–S7 (Supporting Information). Another feature, the peak number of each channel for each window, was also extracted as a feature. Therefore, the feature matrix contained 5 × 16 features for each segmented window. The optimal number of features was determined via REFCV, and then, a random forest algorithm was used for classification. Five‐fold cross‐validation was applied to better utilize the data. The metric recall was used to evaluate the classification performance for each class, and accuracy was used to evaluate the overall performance. The recall of each class and the accuracy of all classes were calculated using Equations S8 and S9 (Supporting Information), as shown in Supporting Information.

### Real‐Time Motion Recognition and Somatosensory Interaction with Virtual Space

For the real‐time somatosensory interaction system, the virtual character and its animations were built using Unity software. The prediction model was a previously trained random forest classifier. The data used for model training were acquired from the subjects when they performed the motions every 3 s, and a fixed window of 3 s was used for the extraction of features (i.e., the average, root mean square, standard deviation, cumulative length, and peak number). To relieve the online computational load, the original 16 channels were fused to eight channels before feature extraction. REFCV was also used to determine the optimal feature number for the classification model. During the real‐time somatosensory interaction process, the motion data containing one complete motion were transmitted to the computer every 3 s. The received data for each time were fed into the prediction model built by random forest, and then, the predicted motion was sent to Unity to control the virtual character to perform the corresponding action.

### Statistical Analysis

All data shown were representative of the samples. The processing procedure of the data is provided in the subsection “Data processing and offline classification.” The sample size for the human test is provided in the subsection “Experiments on human subjects.” The confusion matrices shown in Figures 4 and 5 were normalized by the rows of true labels, with numbers rounded to two decimal places. Data in Figure 2c was shown as mean ± standard deviation. Statistical data were plotted using OriginLab, MATLAB, and Python. More detailed information is provided in the figure captions below.

## Conflict of Interest

The authors declare no conflict of interest.

## Author Contributions

W.J.L. and J.S. conceived the initial concept for creating the sponge‐based sensing array for muscle activities monitoring and somatosensory interaction with virtual space. W.J.L. supervised and guided the project. J.S., Y.L., and J.W. contributed equally to this work. J.S. developed the sensing device and performed the experiments. J.S. and Y.L. performed the data analysis; Y.L. specifically focused on the data using machine learning algorithms and the real‐time controlling system. J.W. (with the supervision of Z.W.) contributed to the background research and introduction. M.C. and Y.L. contributed to the circuit design; M.C. specifically helped develop the wireless data transmission solution. Y.L. and K.W. helped with the human subjects’ test protocols and experiments. X.Y. and N.L. helped with the literature review and Y. K. helped with the illustrations. J.S., Y.L., and W.J.L. co‐drafted this paper. V.A.L., Y.X., W.D., N.L, and Z.W. provided critical technical advice and comments for the experimental studies and helped revise the manuscript.

## Supporting information

Supporting Information

Supplemental Movie 1

## Data Availability

The main data supporting the findings of this study are available within the article and its Supplementary Information. All other relevant source data are available from the corresponding author upon reasonable request.
