# Supplementary material for: AI‐Enabled Soft Sensing Array for Simultaneous Detection of Muscle Deformation and Mechanomyography for Metaverse Somatosensory Interaction
Source: Adv Sci (Weinh). 2024 Feb 20;11(16):2305025. doi: 10.1002/advs.202305025 (PMC11040359; doi:10.1002/advs.202305025)
Supplement: Supplementary file 1 — Supporting Information [file ADVS-11-2305025-s002.pdf]

ADVANCED SCIENCE
Open Access

Supporting Information

for *Adv. Sci.*, DOI 10.1002/advs.202305025

AI-Enabled Soft Sensing Array for Simultaneous Detection of Muscle Deformation and Mechanomyography for Metaverse Somatosensory Interaction

Jiao Suo, Yifan Liu, Jianfei Wang, Meng Chen, Keer Wang, Xiaomeng Yang, Kuanming Yao, Vellaisamy A. L. Roy, Xinge Yu*, Walid A. Daoud, Na Liu, Jianping Wang, Zuobin Wang* and Wen Jung Li**

Supplementary Materials for

AI-enabled Soft Sensing Array for Simultaneous Detection of Muscle Deformation and Mechanomyography for Metaverse Somatosensory Interaction

Jiao Suo, Yifan Liu, Jianfei Wang, Meng Chen, Keer Wang, Xiaomeng Yang, Kuanming Yao, Vellaisamy A. L. Roy, Xinge Yu*, Walid A. Daoud, Na Liu, Jianping Wang, Zuobin Wang*, and Wen Jung Li**

J. Suo, M. Chen, K. Wang, X. Yang, W. Daoud, W. J. Li
Dept. of Mechanical Engineering, City University of Hong Kong, Hong Kong, China
E-mail: cm.chenmeng.1@gmail.com, wenjli@cityu.edu.hk

Y. Liu
Dept. of Electrical and Computer Engineering, Michigan State University, MI, USA

J. Wang, Z. Wang
The Int. Research Centre for Nano Handling and Manufacturing of China, Changchun University of Science and Technology, Changchun, China
E-mail: wangz@cust.edu.cn

K. Yao, X. Yu
Dept. of Biomedical Engineering, City University of Hong Kong, Hong Kong, China
E-mail: xingeyu@cityu.edu.hk

V. A. L. Roy
James Watt School of Engineering, University of Glasgow, Scotland, United Kingdom

N. Liu
Sch. of Mechatronic Engineering and Automation, Shanghai University, China

J. Wang
Dept. of Computer Science, City University of Hong Kong, Hong Kong, China

This PDF file includes:

Supplementary text S1~S4.

Figures S1~S17.

Tables S1~S5.

Other supplementary materials for this manuscript include the following:

Supplementary Movie. Demonstration of the somatosensory interaction application. mp4.

S1. Fabrication process of the sponge-structured sensor and electrode array design

Multiwalled CNTs (MWCNTs) with diameters of 10–20 nm and lengths of 10–30 μm (provided by the manufacturer) were used. First, 0.5 g of MWCNTs were dispersed in sufficient IPA and ultrasonicated for 20 min to obtain a CNT dispersion. Then, 10 g of PDMS base was added to the dispersion, and the mixture was ultrasonicated for 10 min. Subsequently, the mixture was placed on a hotplate (IKA, Germany) maintained at 55°C to evaporate the IPA completely. A PDMS agent (the base-to-agent weight ratio was 10:1) was added to the solution and mechanically mixed. Finally, air bubbles were removed from the mixture through vacuum treatment. The CNT/PDMS solution was then spread on the surface of a sugar cube to obtain the sponge structure. The sugar template was dissolved after the solution was cured in an oven at 70°C for about two h to get the pieces of a thin sponge film with an area of approximately 19.6 mm × 18.4 mm and a thickness of ~400 μm.

For the flexible resistive sensor array, crosstalk is one of the main problems. The main reasons include that the substrates used for the flexible sensor array are usually viscoelastic so the unstressed units may be affected by the deformation of the stressed units; the conductive paths formed by the wires between the sensing units potentially generate the leakage currents in the circuit that create the interface signals in the output reading.[1] Increasing the gap distance between each sensing element in the array is an effective way to suppress the crosstalk phenomenon while it would also reduce the effective occupied area on the entire sensor. In this work, the design of the substrate adapted the hollow-carved design to suppress the interactions among the sensing units by reducing the interaction area of each sensing unit. Each sensing unit was designed to have an individual output wire for signal reading, i.e., using $m * n$ wires (m and n are the number of rows and columns, respectively). Although the simpler circuit structure is to connect all the units in the same row and column and only $m + n$ wires are needed, the errors in reading each resistive element caused by the crosstalk is significant if no additional hardware or correction calculation applied. For example,[2] reported errors of up to 30% for a 4 x 4 array, and [3] 59% for an 8 x 8 array. Considering the space and high resolution are not the primary requirement in the demonstrated application, we chose to apply a 'safe' design, i.e., sufficient wire distance and separated signal output, to ensure the accurate signal reading (the detailed layout is shown in Figure S1c).

S2. Calf muscle and theoretical muscle force estimation theoretically

Human calf mainly consists of two muscles, i.e., the gastrocnemius and the soleus[4]). Gastrocnemius muscle is the largest and most superficial two-joint muscle which connects knee and ankle on the human leg, while soleus is a flat, wide muscle which sits slight deeper than the gastrocnemius, and it only crosses the ankle joint.[5] The gastrocnemius is used extensively during various limb motions since it connects to two joints and it works to flex the ankle and propels humans forward when walking, running, jumping, climbing stairs, etc.[6] The failure of the medial gastrocnemius muscle reduces the peak ankle dorsiflexion angle after landing by 35.5%.[7] So, the gastrocnemius muscle is an ideal muscle for detecting multi-human lower limb motions, and it is also widely used in the sEMG/EMG method to decipher muscle activities.[8-10] As the most superficial and largest muscle of the human calf, gastrocnemius also contributes dominate to the shape of the calf.[11] Therefore, it is appropriate to place the sensor on the gastrocnemius muscle for motion detection and recognition. The gastrocnemius actively participates in the multiple human lower limb motions and its dynamic activities are different when performing different motions. However, although the sensor patch was attached to the position of gastrocnemius, the detected signals are not necessarily only from the gastrocnemius since the sensor patch was attached on the skin surface but not with an intramuscular manner. It is similar to the sEMG which actually detect the muscle group activity from the global skin surface, while intramuscular EMG can detect a single muscle potential by inserting the electrodes into the muscle tissue through the skin.[12] In addition, unlike the sEMG can just detect superficial muscle activity, the mechanomyography (MMG) signals from the deep muscles (e.g., soleus) are possibly to be detected from the skin surface.[13] Therefore, we think the signals detected in this work it is not just gastrocnemius but the lower leg muscle group since the dynamic motions active different muscle.

As for muscle force estimation, Hill's muscle model is one of the most used to study the mechanism of muscle force production, and it is best described as a muscle force (F) with the relationship of muscle shortening velocity (V), as Equation (S1):[14]

$$(F + A)(V + B) = \frac{F_0^m + A}{B} \qquad (S1)$$

where F_0 is the maximum isometric force, A and B are Hill constants. This equation

is under the maximum muscle activation condition. However, research showed that the Hill constant would change with the muscle activation degree.[15] During dynamic muscle motion, muscle activation is time-varying, which can be determined from the neural activation with the specific transformation model. Considering the multiple practical factors, Hill's model-driven muscle force is a function of several parameters (Equation (S2)):[16]

$$F = f(a(t), l^{mt}, l_0^m, l_s^t, \Phi_0, F_0^m) \tag{S2}$$

where $a(t)$ is the time-varying muscle activation, l_o^m is the optimal fiber length, l_s^t is the tendon slack length, Φ_0 is the pennation angle. The parameters except for $a(t)$ would not change during the muscle isometric efforts, but it would be complicated during the dynamic motions. During muscle contraction and expansion, the muscle length would change while its volume remains nearly constant. The muscle's thickness and width also vary with the muscle activation, as shown in Equation (S3).[17] The thickness-to-width ratio (δ) increases with the increased muscle activation (a), while T_0 and W_0 are the thickness and width of the muscle when activation is 0, and λ is the constant coefficient of activation.

$$\delta = (1 + \lambda a)\frac{T_0}{W_0} \tag{S3}$$

Therefore, muscle force estimation is complex during practical dynamic motions. The indirect estimations based on the muscle shape change, EMG and MMG signals, etc., which can be detected on the skin surface and consider the muscle as a 'black box' are effective way.[18]

S3. Effect of body fat on the sensor performance

The effect of the amount of body fat is one of the main concerning issues for the muscle activity measurement. For example, the electrical sEMG signals would suffer attenuation when travelling through the subcutaneous to the skin surface,[19] while the mechanical MMG signals would suffer attenuation too.[20] In addition, the intramuscular fat would limit the muscle fibre's ability to bulge .[21-23] A human subject with the healthy weight BMI and another with the overweight BMI were asked to wear the sensor on their forearm and hold the grip dynamometer tight with the same force (i.e., 5 kg) with his elbow joint and wrist joint fixed. The output signals are shown in Figure S17, and the overweight human subject had a lower response of the sensor, which indicates that the high body fat would cause the degraded sensing performance of the developed muscle sensor. Further improvements (e.g., using an amplifier) should

be made to apply the developed muscle sensor to overweight or obese people.

S4. Equations in the classification process.

The time-domain average, root mean square, standard deviation, and cumulative length of the signal of each channel for each window were calculated with the following Equations (S4)–(S7):

$$\text{Root mean square: } RMS = \sqrt{\frac{1}{N}\sum_{i=1}^{N} X_i^2} \quad (S4)$$

$$\text{Average: } \bar{X} = \frac{1}{N}\sum_{i=1}^{N} X_i \quad (S5)$$

$$\text{Standard deviation: } S.D. = \sqrt{\frac{1}{N}\sum_{i=1}^{N} (X_i - \bar{X})^2} \quad (S6)$$

$$\text{Cumulative length: } WL = \sum_{i=1}^{N-1} |X_{i+1} - X_i| \quad (S7)$$

The recall of each class and the accuracy of all classes were calculated using Equations (S8) and (S9):

$$Recall_j = \frac{N_{TP}(j)}{N_{TP}\,(j) + N_{FN}\,(j)} \quad (S8)$$

$$Accuracy(total) = \frac{N_{TP}(total)}{N_{total}} \quad (S9)$$

where $N_{TP}(j)$ is the number of the true prediction cases of class j, $N_{FN}(j)$ is the number of false negative errors of class j, and N_{total} is the total number of the validation set.

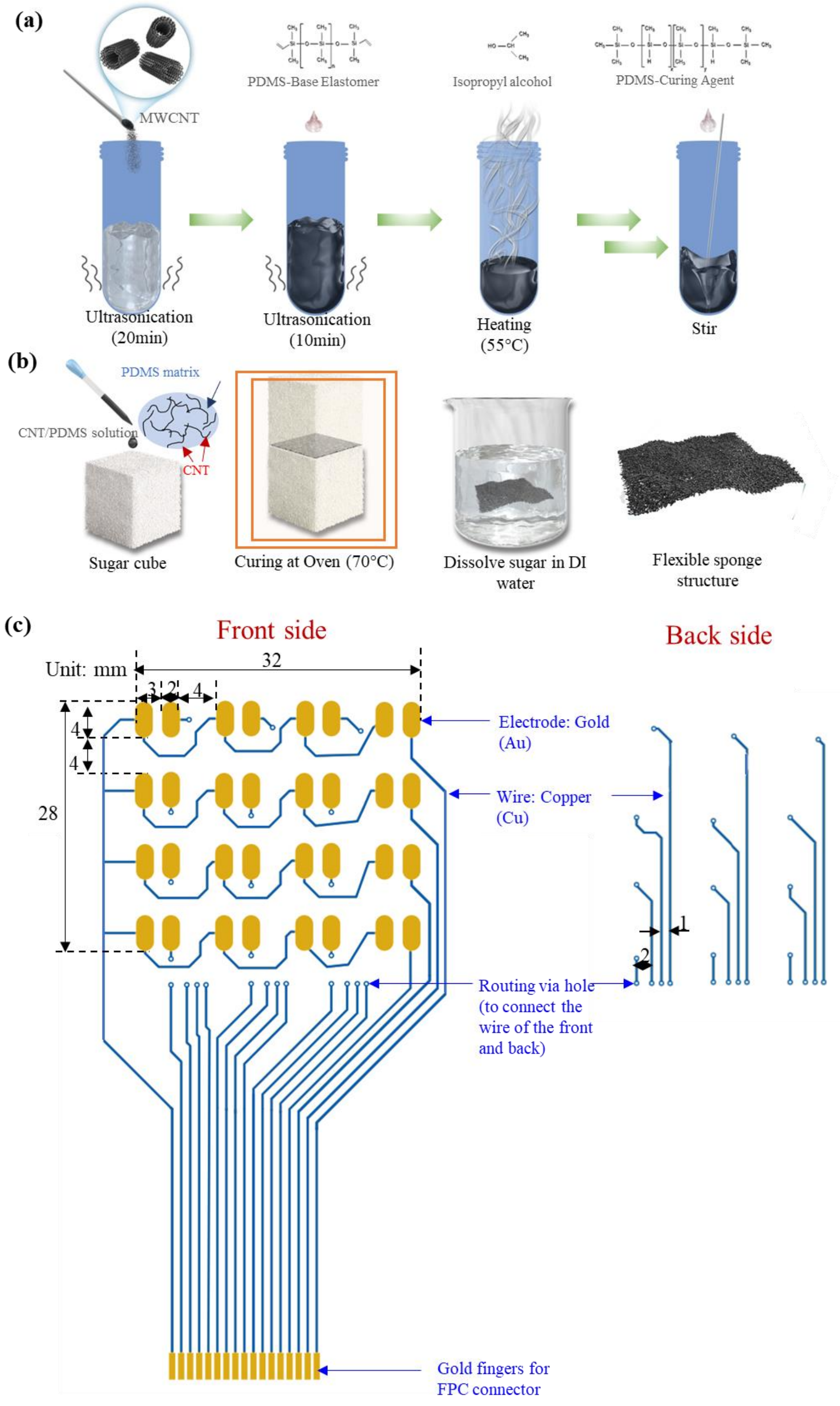

Figure S1. Fabrication process of the CNT/PDMS sponge. (a) Materials synthesis. (b) Sponge structure modeling. (c) The design of the electrode substrate including the front and back side.

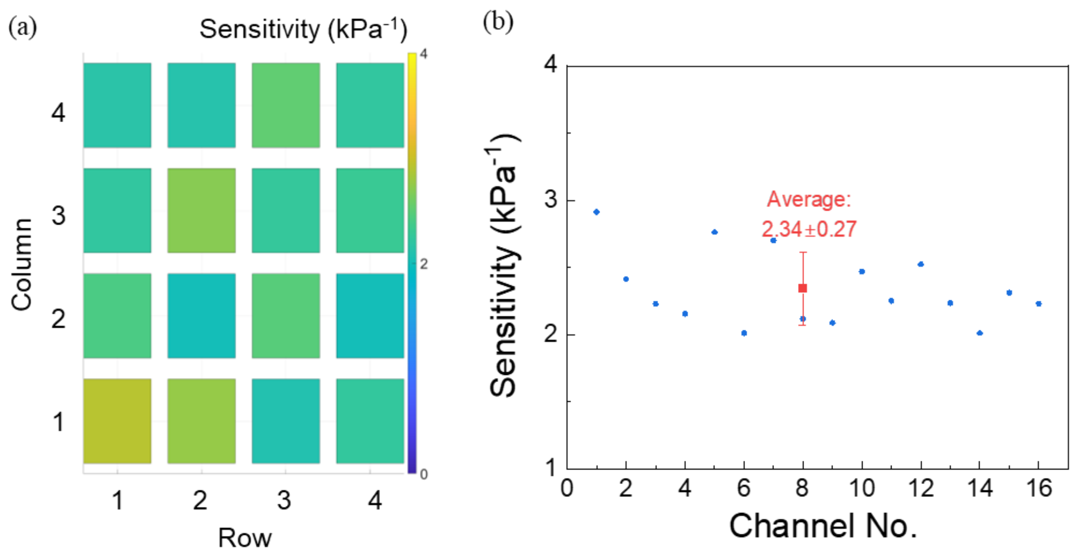

Figure S2. The sensitivity performance (within the pressure range of 0.03-7.8kPa-1) of all the 16 channels in a sensor array.

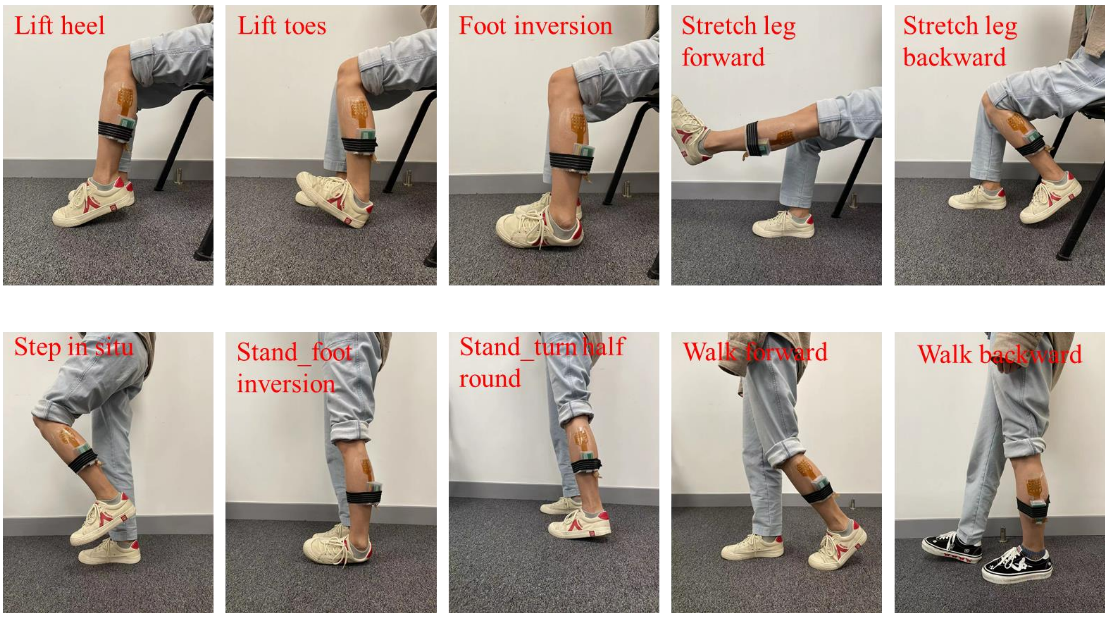

Figure S3. Images of 10 tested lower limb motions.

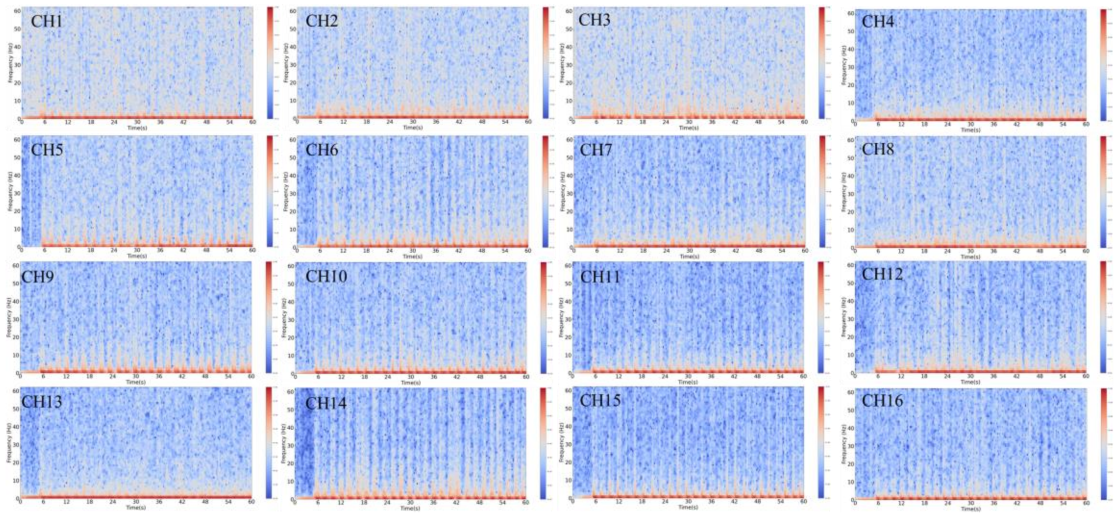

Figure S4. Short-time Fourier transform (STFT) plots of stepping motion (A8) for all the 16 channels.

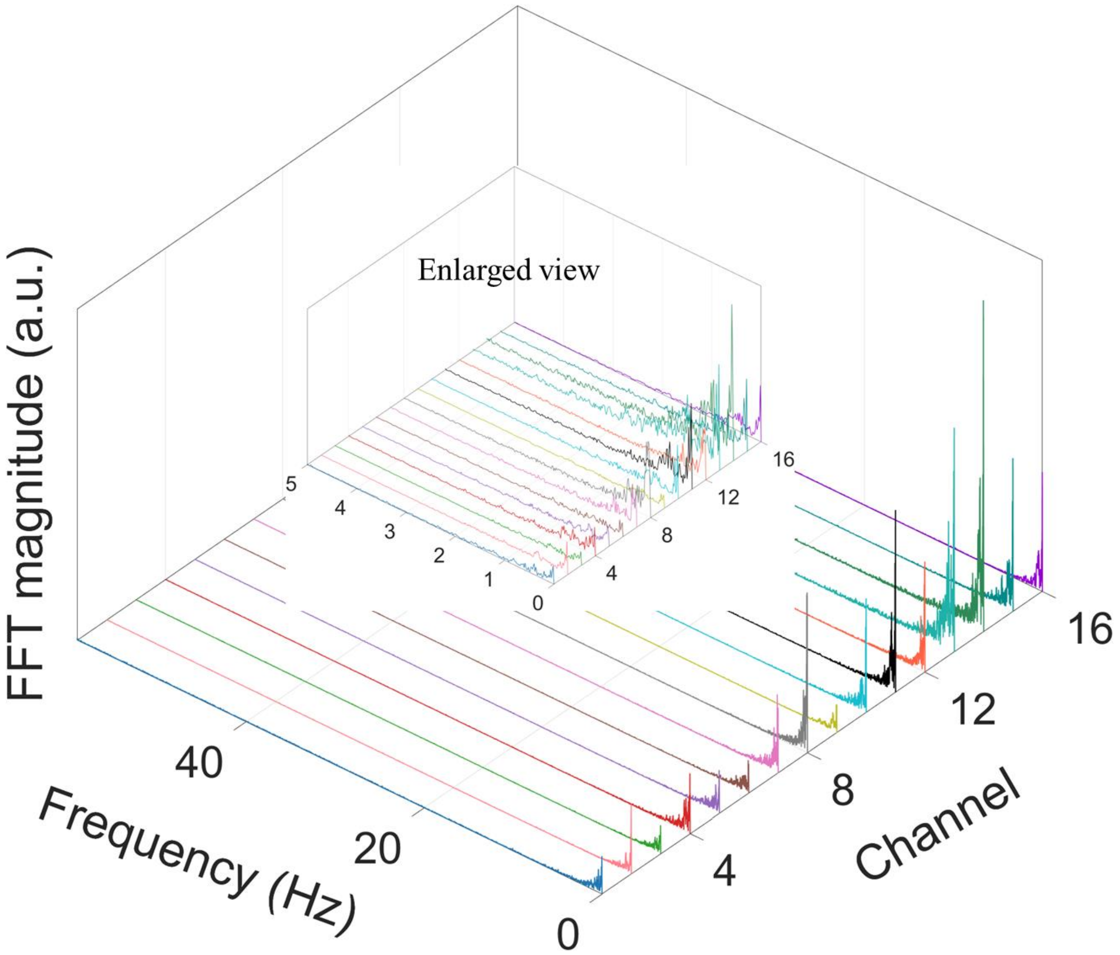

Figure S5. Fast Fourier transform analysis (FFT) on the obtained signals of different motions.

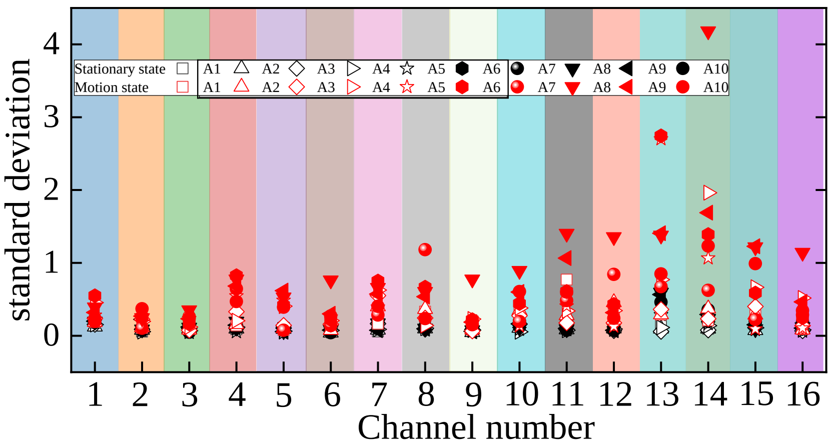

Figure S6. Standard deviations of the output signal change rate of the stationary and in-motion states of A1–A10 from a typical human subject.

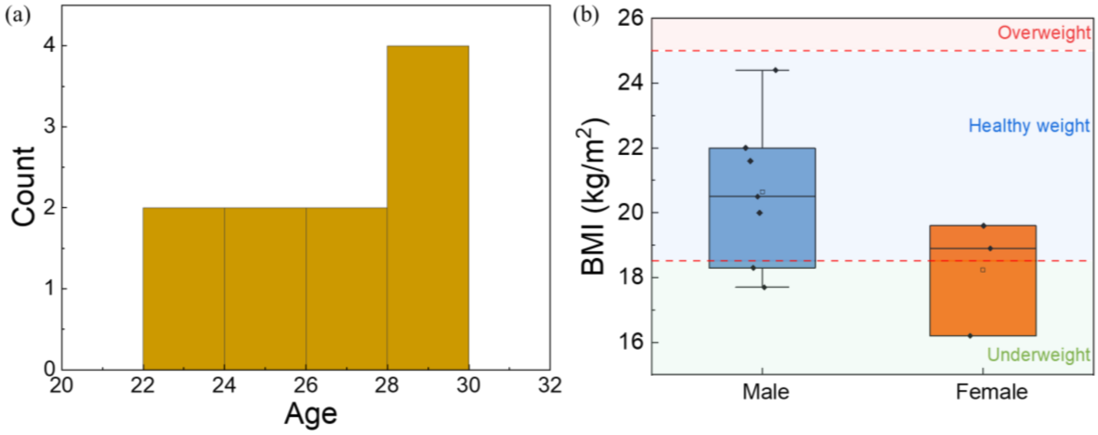

Figure S7. The information of age distribution and body mass index (BMI) of the ten human subjects in this work.

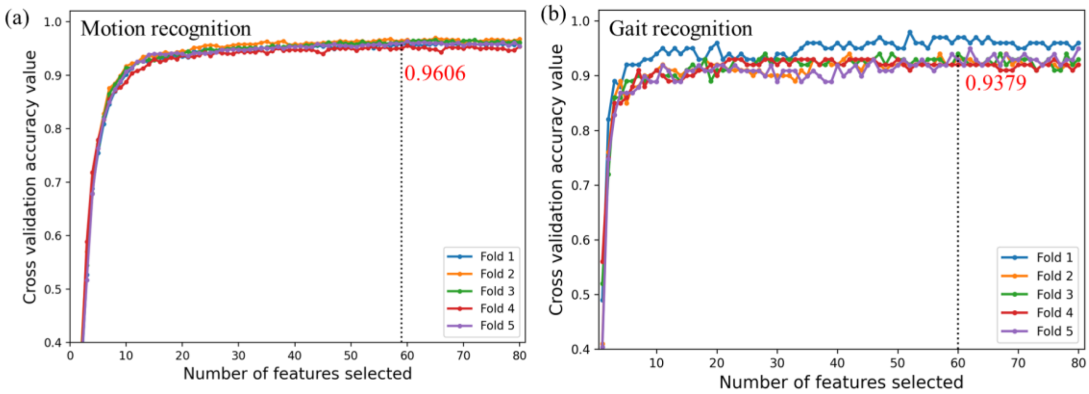

Figure S8. Cross-validation accuracy of the (a) motion and (b) gait recognition with fixed windows.

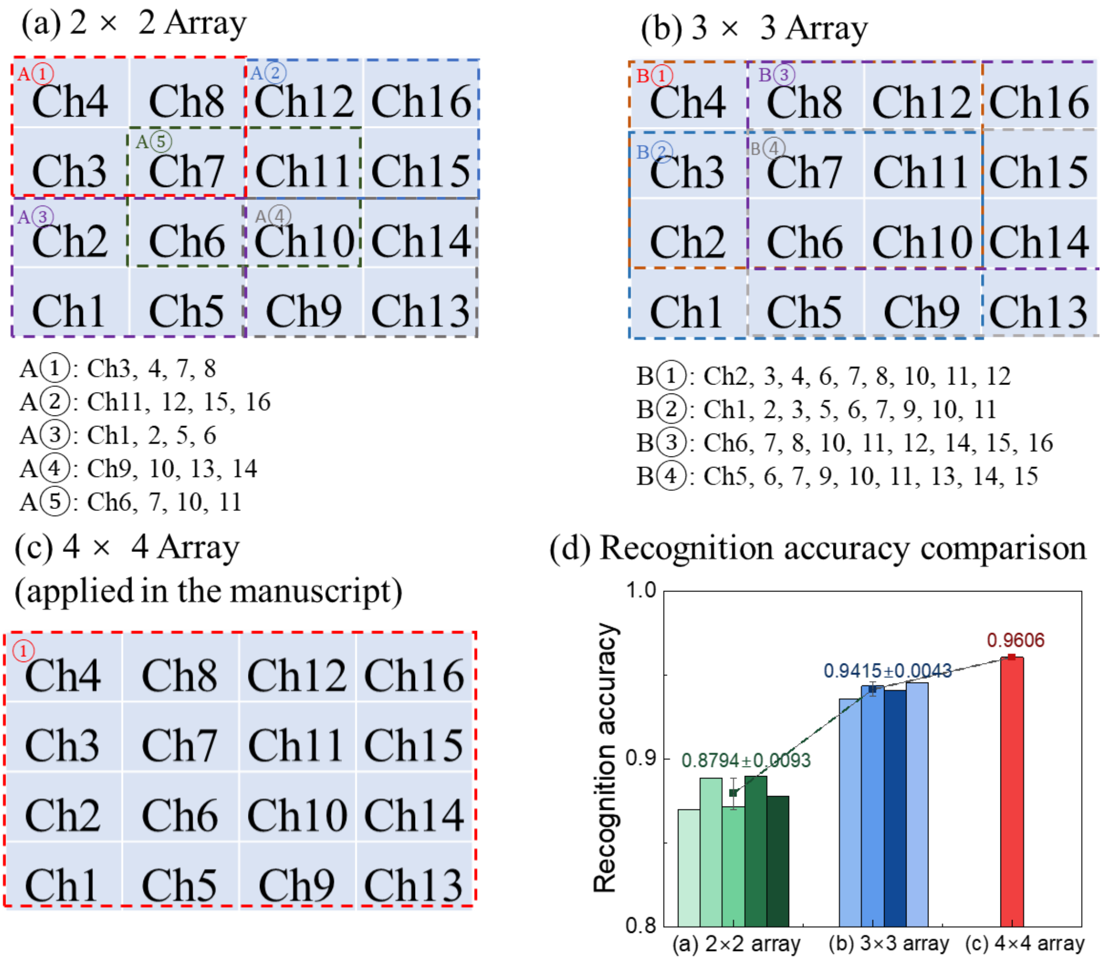

Figure S9. Different size of (a) 2 ×2 and (b) 3 × 3 array extracted from the original (c) 4 × 4 array developed in this work. (d) The comparison of recognition accuracy of using the data of different array size.

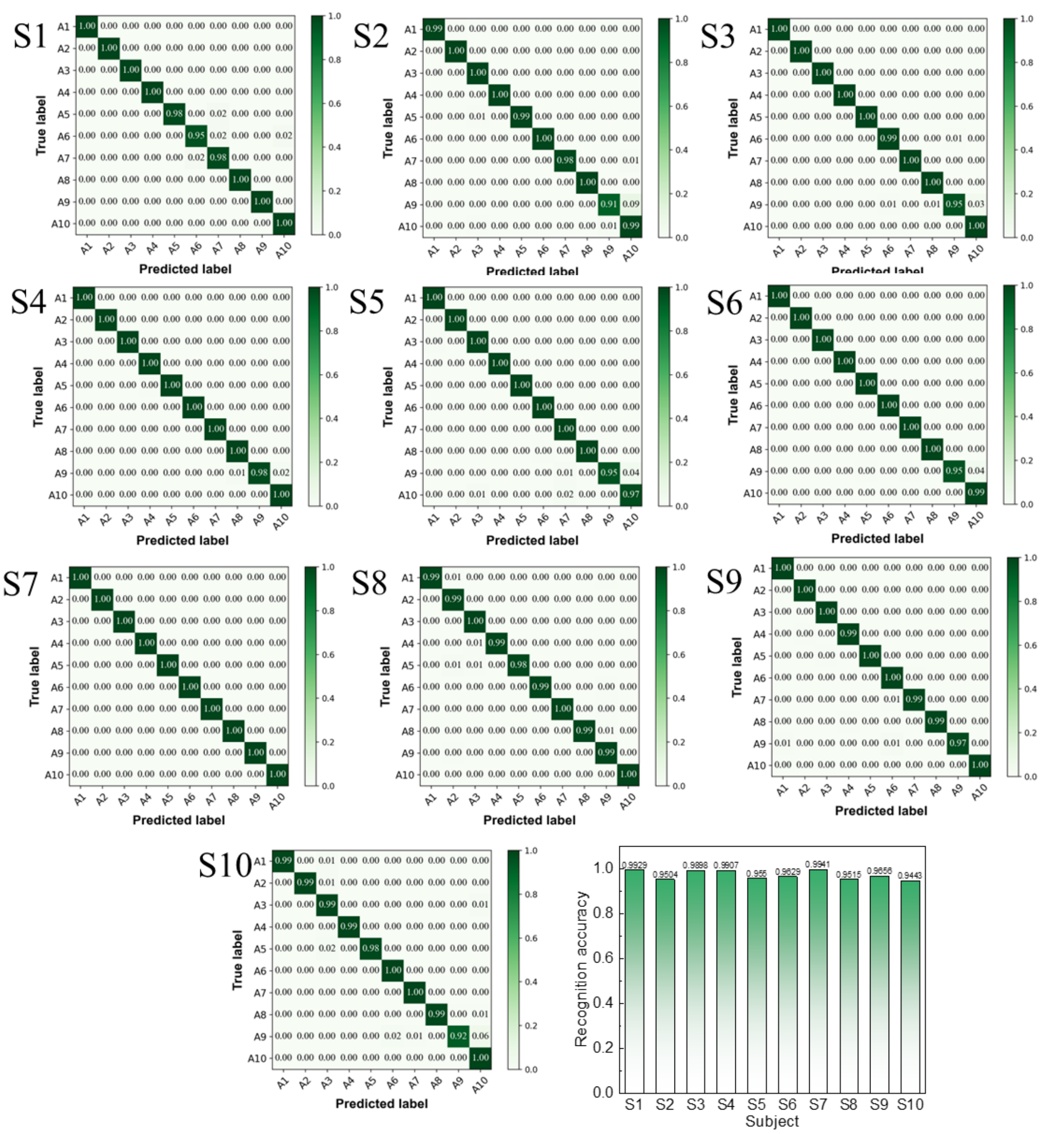

Figure S10. Confusion matrix and recognition accuracy summary of motion recognition results based on the individual subject dataset of ten subjects, respectively.

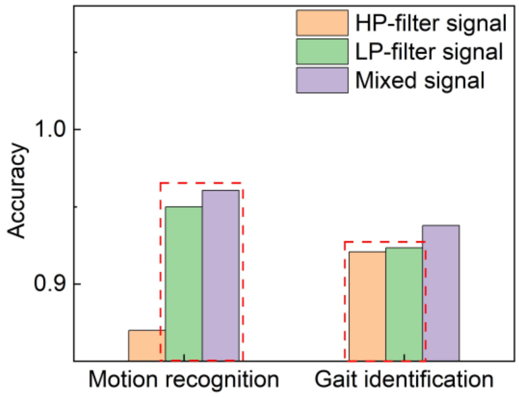

Figure S11. The accuracy of motion recognition and gait identification of using HP-filter, LP-filter, and mixed signals.

(a) The speed of the human subjects S1 and S3 while walking with “slow”, “natural”, and “fast” speeds.

Speed	Human subject S1	Human subject S3
Slow	0.74 m/s	0.85 m/s
Natural	1.12 m/s	1.19 m/s
Fast	1.57 m/s	1.61 m/s

(b) Gait identification results of S1 and S3 with different walking speeds.

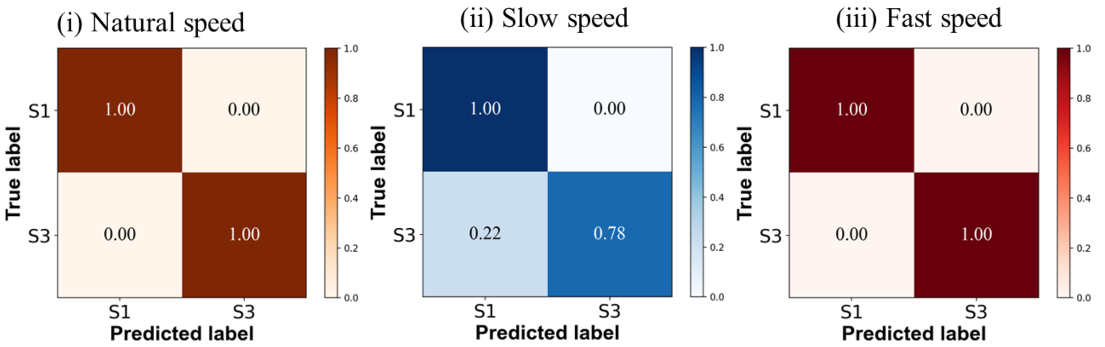

(c) Gait recognition results of slow speed with the improved model.

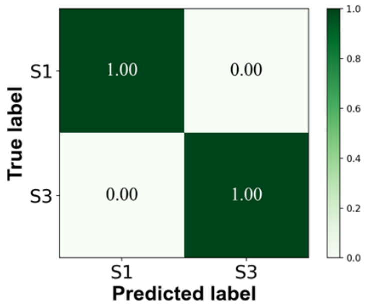

Figure S12. (a) The speed of the human subjects S1 and S3 while walking with “slow”, “natural”, and “fast” speeds. (b) The gait identification results of S1 and S3: (i) walk with natural speed; (ii) walk with slow speed; (iii) walk with fast speed using the model trained in Figure 4(d). (c) Gait recognition results of slow speed with the improved model taking the slow walking data into the model training dataset.

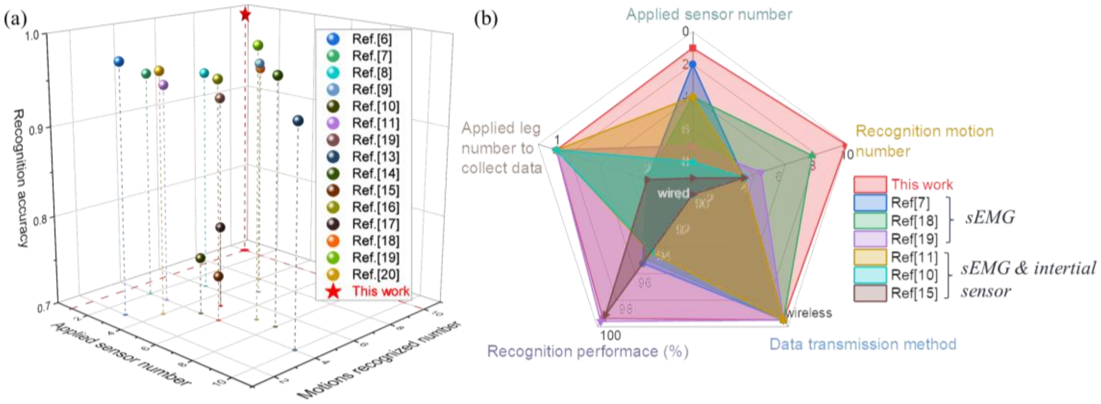

Figure S13. Comparison of the proposed device with other reported EMG/sEMG-based devices for lower limb motion recognition. (a) The number of measured muscles, the number of recognized motions, and recognition accuracy. (b) A radar plot comparing motion recognition performances based on sEMG, sEMG combined with inertial sensors, and the device developed in this study.

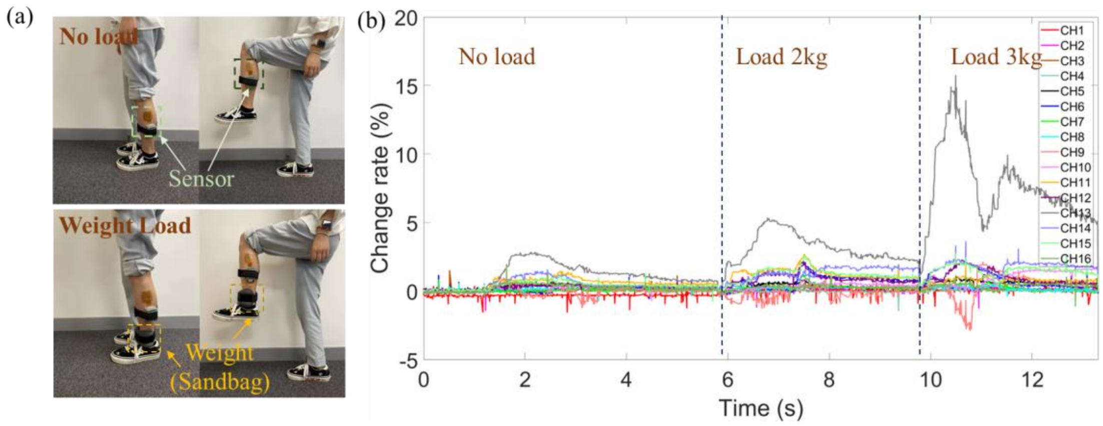

Figure S14. Detection of muscle strength for leg raise with different weights. (a) experimentation picture; (b) output signals.

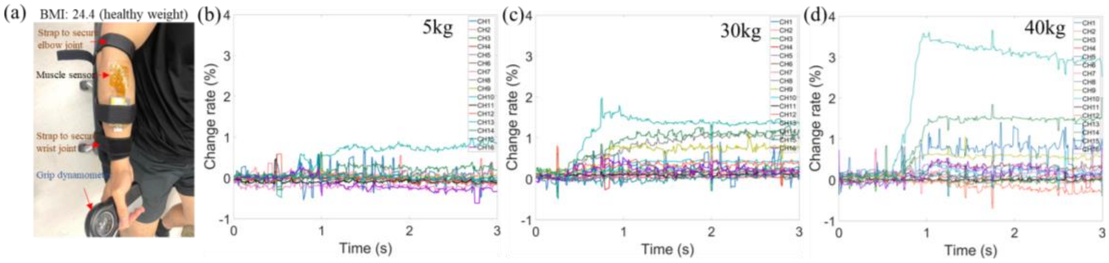

Figure S15. Forearm muscle static contraction with different grip dynamometer reading. (a) The experiment set up. The sensor output signals of the reading of (a) 5 kg, (b) 30 kg, and (c) 40 kg.

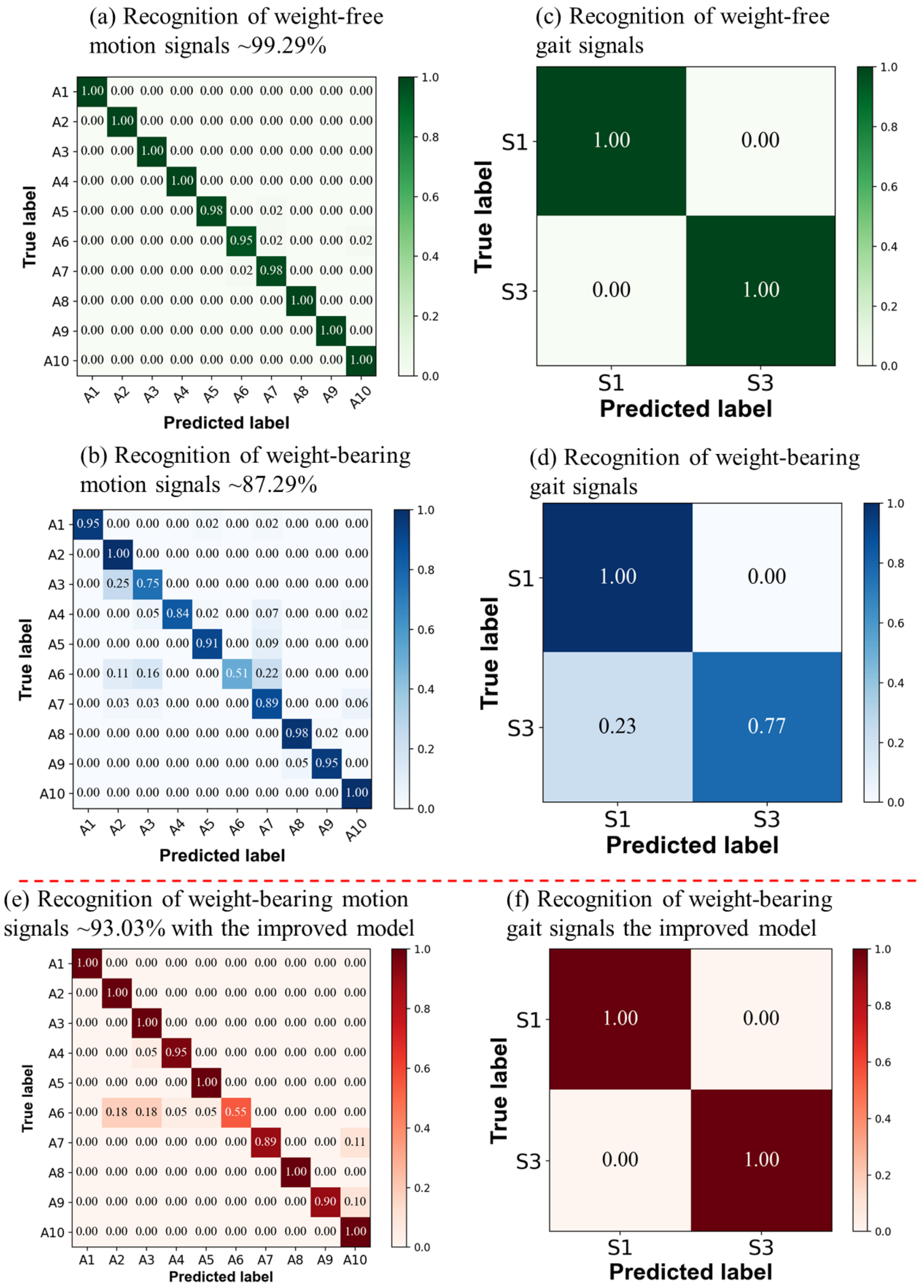

Figure S16. The effect of wearing ankle weight on the motion recognition and gait identification. (a) Recognition accuracy of the weight-free motion signals from S1. (b) Recognition accuracy of the weight-bearing motion signals from S1 using the same model as (a). (c) Gait identification accuracy of weight-free gait signals from S1 and S3. (d) Gait identification accuracy of weight-bearing gait signals from S1 and S3 using the same model as (c). (e) Recognition accuracy of the weight-bearing motion signals from S1 using the improved model with adding the weight-bearing motion signals into the training dataset. (f) Gait identification accuracy of weight-bearing gait signals from S1 and S3 using the improved model with adding the weight-bearing gait data into the training dataset.

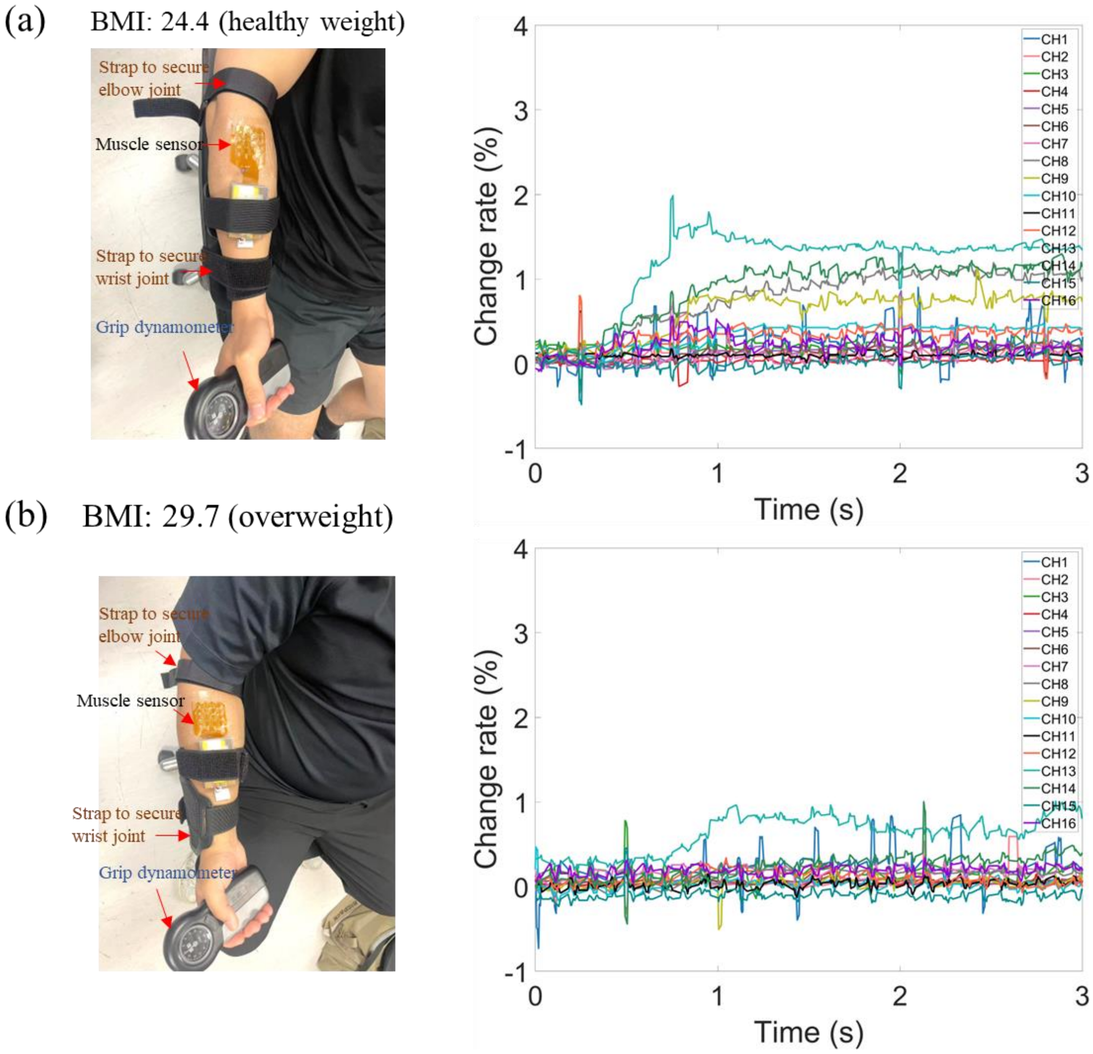

Figure S17. Testing the developed muscle sensing device on a (a) healthy weight and (b) overweight subject by attaching the sensor patch on the forearm.

Table S1. Classification results of motion recognition under different sliding window overlaps.

Overlap	Recall of different motions										Accuracy
	A1	A2	A3	A4	A5	A6	A7	A8	A9	A10	
0	0.96	0.97	0.98	0.96	0.97	0.95	0.96	0.97	0.83	0.94	0.9606
25%	0.97	0.98	0.99	0.97	0.98	0.95	0.97	0.97	0.84	0.95	0.9674
50%	0.98	0.99	0.99	0.98	0.98	0.97	0.98	0.98	0.88	0.98	0.9768
80%	1	1	1	0.99	0.99	0.99	0.99	0.99	0.96	1	0.994

Table S2. Classification results of gait identification under different sliding window overlaps.

Overlap	Recall of different subjects										Accuracy
	S1	S2	S3	S4	S5	S6	S7	S8	S9	S10	
0	0.99	0.98	1	0.79	0.89	0.97	0.95	1	1	0.7	0.9379
25%	1	0.98	1	0.84	0.89	1	0.98	1	0.97	0.6	0.9434
50%	0.99	0.98	1	0.79	0.89	1	1	1	1	0.79	0.9488
80%	1	0.99	1	0.9	0.91	1	0.99	1	1	0.89	0.9683

Table S3. Typical human motion monitoring devices with different sensing mechanisms.

**Motion monitoring devices**	**vision-based non-contact device**[24, 25]	**Wearable device**			
		Angle encoder[26, 27]	Inertial sensors[28, 29]	Muscle sensors	
				EMG/sEMG[30, 31]	Flexible pressure sensor
**Measurement Principle**	Deep learning-based imaging processing	Bending angle of joints	Acceleration and angular velocity	Muscle electrical activity	Muscle mechanical deformation
**Advantages**	Non-contact, long-distance, and highly precise	Data simple and intuitive	Mature techniques and widely used in commercial products	Currently, the standard muscle activity measurement technique.	Lightweight and high sensitivity when using advanced materials; more robust than the weak muscle electrical signals
**Disadvantages**	Requirements in surrounding lighting and environment; high data volume and processing complexity	Not very compliant to human body when attached across joints	Essentially rigid structures that cause discomfort in long-term usage	Sensitive to skin impedance; Amplifier is necessary due to the weak electrical signals	Still in the early stages of development and currently limited to laboratory applications

Table S4. MMG sensors and the related research topics.

Sensor type	Research topic/application	**Remarks** (methods/devices used together with sensors)
Accelerometer	Disease detection, i.e., investigating the applicability in respiratory disease monitoring [32]	sEMG and thoracic bioimpedance
	Muscle force analysis [33, 34]	Force transducer
	Muscle activities with falls and fall-related injuries [35]	EMG
	Gait analysis of stroke survivors [36]	EMG, wearable ultrasonic probe, and thin-film force sensor
	Torque estimation for joints (e.g., elbow [37] and knee [38])	EMG and force signal [38]
	Muscle fatigue analysis [39, 40]	Near-infrared spectroscopy sensors [39]
	Finger movement and grasping gesture recognition [41]	Force-sensitive resistors
	Swallowing activity analysis [42]	_
Condenser microphone	Quantification of motor function post-stroke [43]	IMU
	Analysis of functional muscle connectivity [44]	_
	Joint (knee) torque estimation [45]	EMG
Photoreflector	Muscle contraction performance analysis [46]	EMG
Coupled piezoelectric sensor	Hand motion classification [47]	_
Graphene-based cellular materials	Voluntary bicep muscle contractions [48]	sEMG

Table S5. Typical flexible pressure/strain sensors for human motion detection and recognition.

Ref	Sensing mechanism	Sensor position	Number of motions detected	Subject	Motion recognition accuracy	Muscle deformation	Muscle vibration (MMG)
[49]	Piezoresistive	Back	2 (sitting positions)	1	Not mentioned	Yes	No
[50]	Triboelectric	Biceps brachii	2 (arm movements)	1	Not mentioned	Yes	No
[51]	Piezoresistive	Wrist	12 (hand gestures)	5	96.33%	Yes	No
[52]	Triboelectric + Piezoelectric	Wrist	26 (sign language)	1	92.60%	Yes	No
[53]	Triboelectric + electrostatic	Finger	11 (sign language)	4	98.63%	Yes	No
[54]	Triboelectric + Piezoelectric	Elbow, palm	5 (body movements)	1	82.30%	Yes	No
**This work**	**Piezoresistive**	**Calf muscle**	**10 (lower limb motions)**	**10**	**96.06%**	**Yes**	**Yes**

Supplementary Movie: Demonstration of the somatosensory interaction application. mp4.

References

[1] R. Wang, S. Hu, W. Zhu, Y. Huang, W. Wang, Y. Li, Y. Yang, J. Yu, Y. Deng, *Progress in Natural Science: Materials International* **2023**.

[2] R. Yarahmadi, A. Safarpour, R. Lotfi, *IEEE Sensors Journal* **2015**, *16* (1), 210.

[3] J. A. Hidalgo-López, R. Fernández-Ramos, J. Romero-Sánchez, J. F. Martín-Canales, F. J. Ríos-Gómez, *Journal of Sensors* **2018**, *2018*.

[4] C. Wilson, Gastrocnemius Muscle, https://www.foot-pain-explored.com/gastrocnemius.html, accessed.

[5] Calf Muscle, https://my.clevelandclinic.org/health/body/21662-calf-muscle, accessed.

[6] J. Amis, *Foot and ankle clinics* **2014**, *19* (4), 637.

[7] D. Zeng, H. Wu, X. Zhao, W. Lu, X. Luo, *IEEE Access* **2020**, *8*, 215915.

[8] J. Wang, Y. Dai, T. Kang, X. Si, in *2021 IEEE 4th International Conference on Electronics Technology (ICET)* IEEE, **2021**, 1234-1239.

[9] X. Shi, P. Qin, J. Zhu, S. Xu, W. Shi, *Mathematical Problems in Engineering* **2020**, *2020*.

[10] C. Zhou, L. Yang, H. Liao, B. Liang, X. Ye, *Sensors and Actuators A: Physical* **2021**, *331*, 113025.

[11] A. Sareen, Gastrocnemius, https://www.physio-pedia.com/Gastrocnemius, accessed.

[12] L. Szyszka-Sommerfeld, M. Lipski, K. Woźniak, *Journal of Healthcare Engineering* **2020**, *2020*.

[13] C. S. M. Castillo, S. Wilson, R. Vaidyanathan, S. F. Atashzar, *IEEE Transactions on Neural Systems and Rehabilitation Engineering* **2020**, *29*, 196.

[14] A. V. Hill, *First and last experiments in muscle mechanics*, CUP Archive, **1970**.

[15] J. W. Chow, W. G. Darling, *Journal of Applied Physiology* **1999**, *86* (3), 1025.

[16] K. Manal, T. S. Buchanan, *Journal of biomechanics* **2003**, *36* (8), 1197.

[17] H. Liu, X. Zhang, K. Zhu, H. Niu, in *2021 IEEE Asia Conference on Information Engineering (ACIE)* IEEE, **2021**, 27-31.

[18] D. Wang, Doctoral thesis, University of Science and Technology of China **2019**.

[19] C. Nordander, J. Willner, G.-Å. Hansson, B. Larsson, J. Unge, L. Granquist, S. Skerfving, *European journal of applied physiology* **2003**, *89*, 514.

[20] E. M. Scheeren, L. Mineiro, E. B. Neves, E. Krueger, G. N. Nogueira Neto, P. Nohama, *Research on Biomedical Engineering* **2017**, *32*, 307.

[21] H. Rahemi, N. Nigam, J. M. Wakeling, *Journal of The Royal Society Interface* **2015**, *12* (109), 20150365.

[22] N. Y. Kelp, C. J. Clemente, K. Tucker, F. Hug, S. Pinel, T. J. Dick, *Journal of Applied Physiology* **2023**, *134* (6), 1520.

[23] S. Pinel, N. Y. Kelp, J. M. Bugeja, B. Bolsterlee, F. Hug, T. J. Dick, *Experimental Gerontology* **2021**, *156*, 111594.

[24] T. B. Moeslund, A. Hilton, V. Krüger, *Computer vision and image understanding* **2006**, *104* (2-3), 90.

[25] Ł. Kidziński, B. Yang, J. L. Hicks, A. Rajagopal, S. L. Delp, M. H. Schwartz, *Nature communications* **2020**, *11* (1), 1.

[26] G. Roy, A. Bhuiya, A. Mukherjee, S. Bhaumik, *Procedia computer science* **2018**, *133*, 763.

[27] X. Zou, X. Li, J. Xue, K. W. C. Lai, in *2023 IEEE 18th International Conference on Nano/Micro Engineered and Molecular Systems (NEMS)* IEEE, **2023**, 161-164.

[28] S. Liu, J. Zhang, Y. Zhang, R. Zhu, *Nature communications* **2020**, *11* (1), 1.

[29] M. O'Reilly, B. Caulfield, T. Ward, W. Johnston, C. Doherty, *Sports Medicine* **2018**, *48* (5), 1221.

[30] G. Ray, S. Guha, *IEEE Transactions on Biomedical Engineering* **1983**, (2), 130.
[31] N. Kim, T. Lim, K. Song, S. Yang, J. Lee, *ACS applied materials & interfaces* **2016**, *8* (32), 21070.
[32] D. Blanco-Almazan, W. Groenendaal, M. Lozano-Garcia, L. Estrada-Petrocelli, L. Lijnen, C. Smeets, D. Ruttens, F. Catthoor, R. Jané, *IEEE Transactions on Biomedical Engineering* **2020**, *68* (1), 298.
[33] E. Cè, G. Coratella, C. Doria, M. Borrelli, S. Rampichini, E. Limonta, S. Longo, F. Esposito, *European Journal of Applied Physiology* **2022**, 1.
[34] Z. Li, L. Gao, W. Lu, D. Wang, H. Cao, G. Zhang, *Sensors* **2022**, *22* (12), 4651.
[35] R. T.-L. Zhu, P.-Z. Lyu, S. Li, C. Y. Tong, Y. T. Ling, C. Z.-H. Ma, *Biosensors* **2022**, *12* (6), 430.
[36] P.-Z. Lyu, R. T.-L. Zhu, Y. T. Ling, L.-K. Wang, Y.-P. Zheng, C. Z.-H. Ma, *Biosensors* **2022**, *12* (5), 349.
[37] Y. Shi, W. Dong, W. Lin, L. He, X. Wang, P. Li, Y. Gao, *Electronics* **2022**, *11* (9), 1335.
[38] N. Hondo, T. Tsuji, *IEEE Transactions on Neural Systems and Rehabilitation Engineering* **2022**, *30*, 1120.
[39] N. S. Mohamad Saadon, N. A. Hamzaid, N. Hasnan, M. A. Dzulkifli, M. Teoh, G. M. Davis, *Artificial Organs* **1997**.
[40] M. R. M. Ismail, C. K. Lam, K. Sundaraj, M. H. F. Rahiman, *Journal of Musculoskeletal & Neuronal Interactions* **2021**, *21* (4), 481.
[41] S. B. Moqadam, A. S. Asheghabadi, J. Xu, *IEEE Sensors Journal* **2021**, *22* (3), 2591.
[42] A. Mialland, B. Kinsiklounon, G. Tian, C. Noûs, A. Bonvilain, *IRBM* **2021**.
[43] L. Formstone, W. Huo, S. Wilson, A. McGregor, P. Bentley, R. Vaidyanathan, *IEEE Transactions on Neural Systems and Rehabilitation Engineering* **2021**, *29*, 1158.
[44] C. S. M. Castillo, R. Vaidyanathan, S. F. Atashzar, *IEEE Transactions on Biomedical Engineering* **2022**.
[45] C. Caulcrick, W. Huo, W. Hoult, R. Vaidyanathan, *IEEE Robotics and Automation Letters* **2021**, *6* (4), 7185.
[46] S. Fukuhara, T. Kawashima, H. Oka, *Scientific Reports* **2021**, *11* (1), 1.
[47] M. Szumilas, M. Władziński, K. Wildner, *Sensors* **2021**, *21* (24), 8380.
[48] Z. He, Z. Qi, H. Liu, K. Wang, L. Roberts, J. Z. Liu, Y. Liu, S. J. Wang, M. J. Cook, G. P. Simon, *National Science Review* **2022**, *9* (4), nwab184.
[49] Y. Wei, X. Shi, Z. Yao, J. Zhi, L. Hu, R. Yan, C. Shi, H.-D. Yu, W. Huang, *npj Flexible Electronics* **2023**, *7* (1), 13.
[50] C. Wang, X. Qu, Q. Zheng, Y. Liu, P. Tan, B. Shi, H. Ouyang, S. Chao, Y. Zou, C. Zhao, *ACS nano* **2021**, *15* (6), 10130.
[51] Y. Liu, X. Liang, H. Li, H. Deng, X. Zhang, D. Wen, M. Yuan, H. Heidari, R. Ghannam, X. Zhang, *Advanced Intelligent Systems* **2022**, *4* (11), 2200193.
[52] P. Tan, X. Han, Y. Zou, X. Qu, J. Xue, T. Li, Y. Wang, R. Luo, X. Cui, Y. Xi, *Advanced Materials* **2022**, *34* (21), 2200793.
[53] Z. Zhou, K. Chen, X. Li, S. Zhang, Y. Wu, Y. Zhou, K. Meng, C. Sun, Q. He, W. Fan, *Nature Electronics* **2020**, *3* (9), 571.
[54] M. H. Syu, Y. J. Guan, W. C. Lo, Y. K. Fuh, *Nano Energy* **2020**, *76*, 105029.
[53] M. S. Al-Quraishi, I. Elamvazuthi, S. A. Daud, S. Parasuraman, A. Borboni, in *2018 International Conference on Intelligent and Advanced System (ICIAS)* IEEE, **2018**, 1-5.
[54] M. Zhou, C. Ren, H. Liu, in *2019 IEEE 3rd Advanced Information Management, Communicates,*

Electronic and Automation Control Conference (IMCEC) IEEE, **2019**, 21-25.
[55] J. Wang, L. Wang, X. Xi, S. M. Miran, A. Xue, *Electronics* **2020**, *9* (4), 556.
[56] B. Zhou, H. Wang, F. Hu, N. Feng, H. Xi, Z. Zhang, H. Tang, *Computer Methods and Programs in Biomedicine* **2020**, *193*, 105486.
[57] J. Wang, D. Cao, J. Wang, C. Liu, *Sensors* **2021**, *21* (18), 6147.
[58] J. Wang, Y. Dai, X. Si, *Electronics* **2021**, *10* (20), 2473.
[59] X. Zhang, S. Tao, *Wireless Communications and Mobile Computing* **2022**, *2022*.
[60] Y. Wang, X. Cheng, L. Jabban, X. Sui, D. Zhang, *IEEE Sensors Journal* **2022**.
[61] J. Liu, C. Wang, B. He, P. Li, X. Wu, *IEEE Transactions on Medical Robotics and Bionics* **2022**.
[62] S. Kyeong, J. Feng, J. K. Ryu, J. J. Park, K. H. Lee, J. Kim, *International Journal of Control, Automation and Systems* **2022**, *20* (3), 1018.
[63] Z. Peng, Z. Junxia, A. M. M. Elsabba, *IEEE Sensors Journal* **2022**.
[64] C. Wei, H. Wang, Y. Lu, F. Hu, N. Feng, B. Zhou, D. Jiang, Z. Wang, *Biomedical Signal Processing and Control* **2022**, *71*, 103198.
[65] A. R. Acosta, in *2021 IEEE Fifth Ecuador Technical Chapters Meeting (ETCM)* IEEE, **2021**, 1-6.
